# The influence of the axial group on the crystal structures of boron sub­phthalo­cy­an­ines

**DOI:** 10.1107/S2053229624006934

**Published:** 2024-09-04

**Authors:** Rachel Zigelstein, Alan J. Lough, Timothy P. Bender

**Affiliations:** a Department of Chemical Engineering and Applied Chemistry, University of Toronto, 200 College Street, Toronto, Ontario, M5S 3E5, Canada; b Department of Chemistry, University of Toronto, 80 St. George Street, Toronto, Ontario, M5S 3H6, Canada; c Department of Materials Science and Engineering, University of Toronto, 184 College Street, Toronto, Ontario, M5S 3E4, Canada; d Department of Mechanical & Industrial Engineering, University of Toronto, 5 King’s College Road, Toronto, Ontario, M5S 3G8, Canada; Universidade Federal de Minas Gerais, Brazil

**Keywords:** crystal structure, subphthalocyanine, boron, axial, crystal growth, solid state, sublimation, slow vapour diffusion, slow evaporation, organic electronics

## Abstract

The crystal structures of 16 boron sub­phthalo­cy­an­ines (BsubPcs) with structurally diverse axial groups were analyzed and com­pared to elucidate the impact of the axial group on the inter­molecular π–π inter­actions, axial-group inter­actions, axial bond length and BsubPc bowl depth. π–π inter­actions between the iso­indole units of adjacent BsubPc mol­ecules most often involve concave–concave packing, whereas axial-group inter­actions with adjacent BsubPc mol­ecules tend to favour the convex side of the BsubPc bowl. Furthermore, axial groups that contain O and/or F atoms tend to have significant hy­dro­gen-bonding inter­actions, while axial groups containing arene site(s) can participate in π–π inter­actions with the BsubPc bowl, both of which can strongly influence the crystal packing. Bulky axial groups did tend to disrupt the π–π inter­actions and/or axial-group inter­actions, preventing some of the close packing that is seen in BsubPcs with less bulky axial groups. The atomic radius of the heteroatom bonded to boron directly influences the axial bond length, whereas the axial group has minimal impact on the BsubPc bowl depth. Finally, the crystal growth method did not generally appear to have a significant impact on the solid-state arrangement, with the exception of water occasionally being incorporated into crystal structures when hygroscopic solvents were used. These insights can help with the design and fine-tuning of the solid-state structures of BsubPcs as they continue to be developed as functional materials in organic electronics.

## Abstract 2

The crystal structures of an array of 16 boron sub­phthalo­cy­an­ines with structurally diverse axial groups were analyzed and com­pared based on their inter­molecular inter­actions, axial bond lengths and bowl depths.

## Introduction

**Figure 1:**
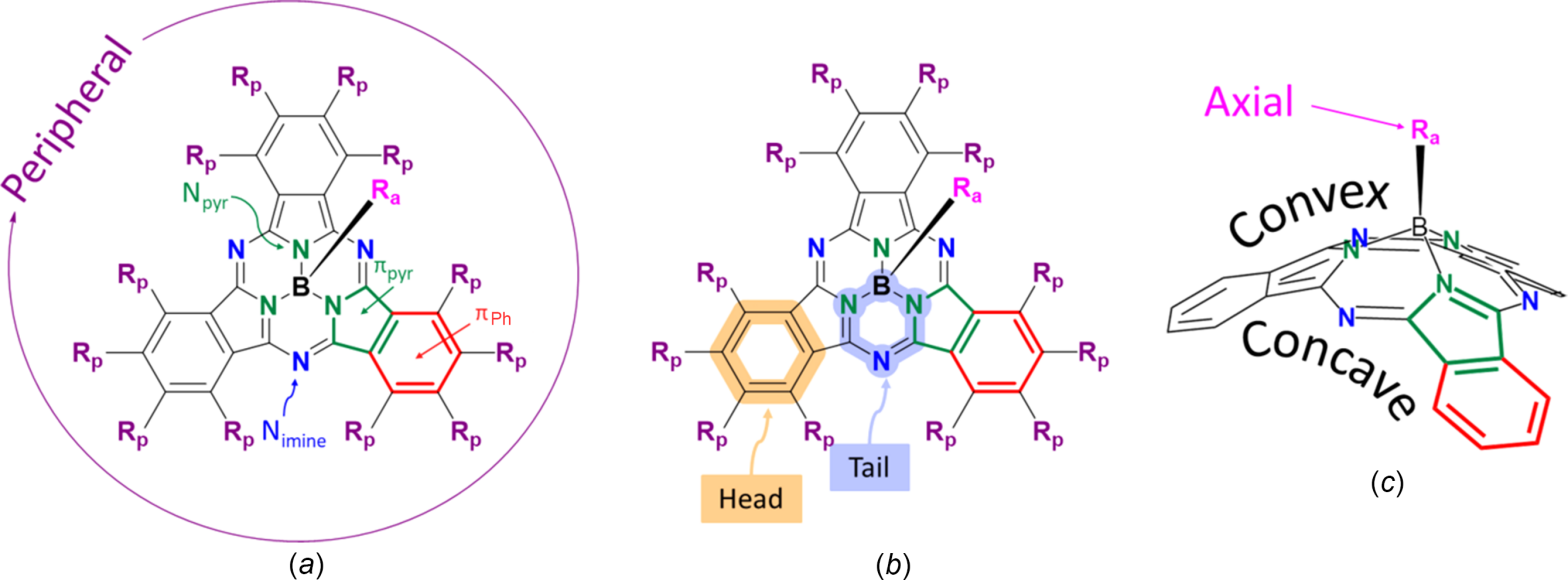
(*a*) General 2D structure of the BsubPc unit with key terms labelled, including the peripheral substituents (*R*_p_), imine (N_imine_) and pyrrole (N_pyr_) N atoms, and different π units (π_Ph_ in red and π_pyr_ in green). (*b*) General 2D structure of the BsubPc unit with ‘head’ (orange highlight) and ‘tail’ (purple highlight) labelled. (*c*) General 3D structure of the BsubPc unit with the axial substituent (*R*_a_) and concave/convex sides of the bowl labelled. The peripheral substituents have been omitted from the 3D structure for clarity.

Boron sub­phthalo­cy­an­ines (BsubPcs) are a class of nonplanar aromatic organic com­pounds with a unique bowl-shaped structure. The BsubPcs are homologues of the phthalocyanine (Pc) family and are com­prised of three di­imino­iso­indole units templated around a boron chelation centre (Fig. 1 ▸) (Claessens *et al.*, 2014 ▸). An additional substituent bonded to boron protrudes from the convex side of the bowl and is known as the axial group (Farac *et al.*, 2023 ▸; Zigelstein & Bender, 2024 ▸). BsubPcs were first synthesized accidentally by Meller and Ossko in 1972 when trying to incorporate boron as the central atom in a phthalocyanine (Pc) (Grant *et al.*, 2019 ▸; Meller & Ossko, 1972 ▸). Instead of the anti­cipated planar macrocycle with four repeating di­imino­iso­indole units that is a part of the Pc structure, they discovered Cl-BsubPc as a purple nonplanar com­pound with three di­imino­iso­indole units around the boron centre, with chlorine in the axial position [Fig. 1 ▸(*c*)] (Grant *et al.*, 2019 ▸). The characteristic nonplanar shape of BsubPcs is caused by the atomic radius of boron being slightly larger than the cavity it occupies (Morse & Bender, 2012*a* ▸). Numerous BsubPc derivatives have been synthesized in the ∼50 years since their discovery, with many physical properties, including the solid-state arrangement, being influenced by substitution at the axial and/or peripheral positions of the BsubPc macrocycle (Morse *et al.*, 2014 ▸). The unique optical, electronic and physical properties of BsubPcs, as well as the ability to tune these properties through axial and/or peripheral derivatization, have led to the development of these materials for organic electronic applications, such as organic photovoltaics (OPVs) (Grant *et al.*, 2019 ▸; Mutolo *et al.*, 2006 ▸), organic light-emitting diodes (OLEDs) (Morse & Bender, 2012*a* ▸: Morse *et al.*, 2010 ▸) and organic thin film transistors (OTFTs) (Yasuda & Tsutsui, 2006 ▸).

BsubPcs are typically employed in the solid state as photoactive materials in organic electronics, sparking the inter­est in using X-ray diffraction (XRD) to study the inter­molecular inter­actions that impact their solid-state arrangements (Virdo *et al.*, 2016 ▸). The solid-state arrangement of BsubPcs can be described by the mol­ecular fragment inter­actions between neighbouring BsubPcs and the relative alignment of the isoindoline fragments, with π–π inter­actions commonly being seen between adjacent BsubPcs in the crystal structure (Bukuroshi *et al.*, 2019 ▸). These inter­actions can provide information on the charge-transfer properties of BsubPcs, which is important for their application as organic semiconductors (Castrucci *et al.*, 2012 ▸).

**Figure 2:**
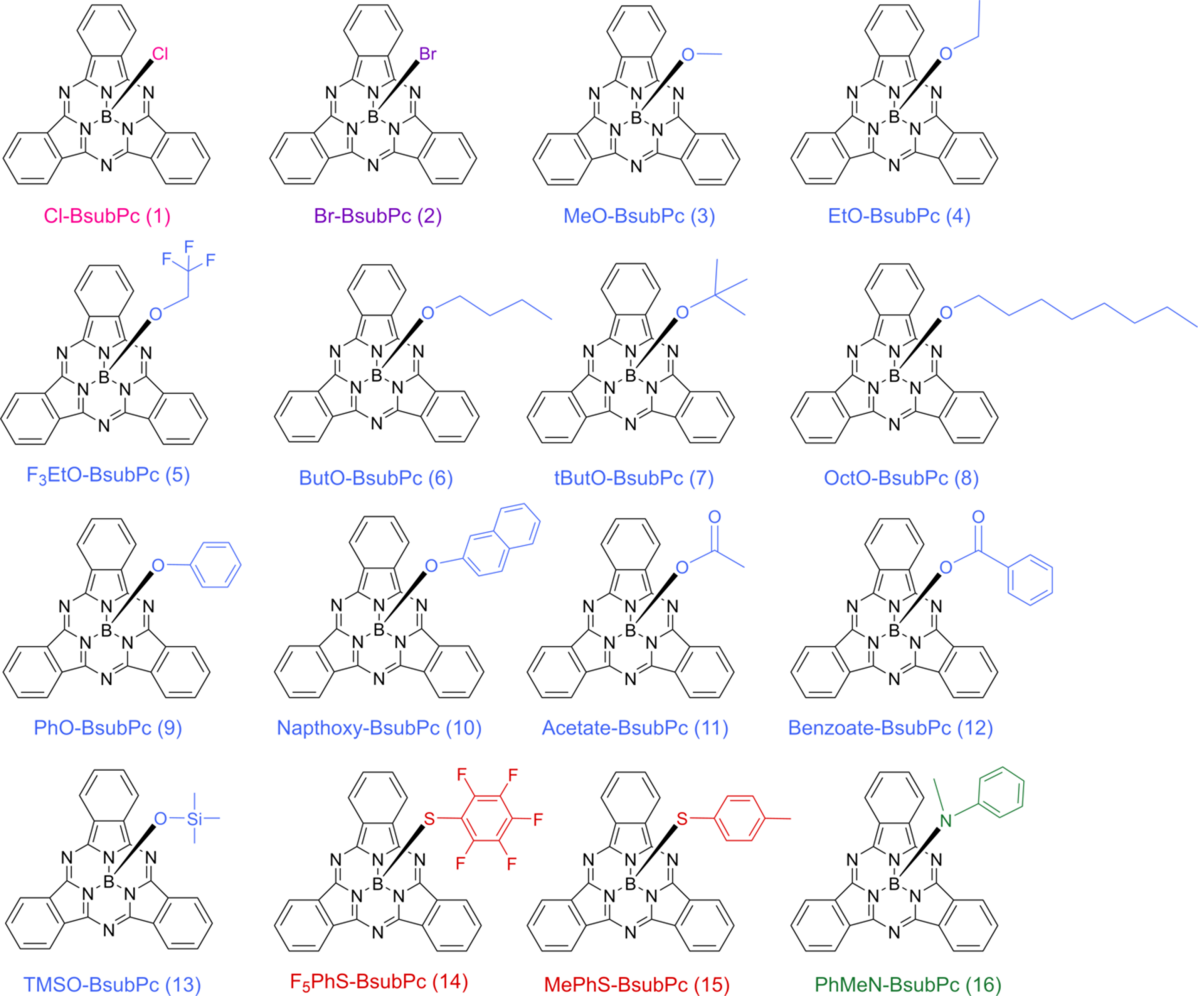
2D structures of investigated axially substituted BsubPcs with the colour of the axial group representing the heteroatom bonded to boron, *i.e.* chlorine – pink, bromine – purple, oxygen – blue, sulfur – red and nitro­gen – green.

In this study, single crystals were grown of an array of BsubPcs with structurally diverse axial groups (Fig. 2 ▸) to investigate how the axial group impacts the π–π inter­actions of BsubPcs in the solid state and explore the inter­molecular inter­actions that involve the axial group. The influence of the axial group on the BsubPc bowl depth and axial bond length is also of inter­est. While crystal structures of some of the BsubPcs investigated in this study have been reported previously, past studies have largely focused on smaller subsets of axial substituents, such as halide (Fulford *et al.*, 2012 ▸), phen­oxy (Paton *et al.*, 2011 ▸) or sulfonate (Paton *et al.*, 2012 ▸) derivatives, but different functional groups are rarely com­pared to each other. A review paper from Lavarda *et al.* (2022 ▸) provides a general summary of the crystal behaviour of axial halide, aryl and ar­yloxy derivatives, but the discussion does not extend to other boron–oxygen-bonded derivatives or include any boron–sulfur- or boron–nitro­gen-bonded derivatives, and the crystals are from multiple laboratories. Therefore, herein, we provide the first report of a systematic com­parison of BsubPc crystal structures with a com­prehensive set of axial substituents, including halide (**1** and **2**), alk­oxy (**3**–**8**), phen­oxy (**9** and **10**), carboxyl­ate (**11** and **12**), sil­oxy (**13**), thio­phen­oxy (**14** and **15**) and amino (**16**) derivatives. Furthermore, we report new polymorphs of some BsubPcs and use different crystal growth methods than have been reported previously.

## Experimental

### Materials and instrumentation

Toluene, heptane, acetone, di­chloro­methane (DCM), hexa­nes, tetra­hydro­furan (THF) and pentane were purchased from Caledon Laboratories (Caledon, Ontario, Canada) and used as received. All X-ray crystal structure data were collected using a Bruker Kappa APEX DUO CMOS Photon II dif­frac­tom­eter. Experimental and refinement details are provided in Table 1 ▸. Structural analysis was conducted using *Mercury* software (Macrae *et al.*, 2020 ▸).

### Synthesis and crystal growth

Briefly, with the exception of Br- and Cl-BsubPc, all other axially substituted BsubPcs were synthesized through an axial exchange reaction with Br-BsubPc and the appropriate nucleophile. In general, the axial exchange reactions were run in anhydrous chloro­benzene with 5–10 equivalents of the nucleophile at 75 °C until all of the Br-BsubPc was consumed, as monitored by high-performance liquid chromatography (HPLC). Full detailed synthetic procedures and characterization by ^1^H NMR and ^19^F NMR spectroscopy, direct analysis in real-time high-resolution mass spectrometry (DART-HRMS) and HPLC for the BsubPcs analysed within this study are provided in a recent publication (Zigelstein & Bender, 2024 ▸).

The train sublimation apparatus that was used to grow several of the crystals analysed in this study has been described previously (Morse *et al.*, 2010 ▸; Virdo *et al.*, 2016 ▸). For crystals grown by slow vapour diffusion of heptane into toluene, 5–10 mg of material was dissolved in toluene in a 20 ml scintillation vial. The vial was then suspended in a sealed jar containing ∼150 ml of heptane and left undisturbed for 2–4 weeks until crystals were seen. For crystals grown by slow evaporation of a common organic solvent (*i.e.* acetone or methanol), 5–10 mg of material was dissolved in solvent in a 20 ml scintillation vial. The vial was then covered with aluminium foil with 3–5 holes poked through and left undisturbed until crystals were seen. For crystals grown during a two-solvent recrystallization, the material was dissolved in THF, and pentane was added dropwise until crystals formed out of solution. These crystals were then collected by gravity filtration. Finally, some crystals formed after rotary evaporation to remove DCM/hexa­nes after purification by column chromatography on standard basic alumina. The crystal growth method used for each com­pound is provided in Table 1 ▸.

### Refinement

Crystal data, data collection and structure refinement details are summarized in Table 1 ▸.

## Results and discussion

Single crystals of 16 axially substituted BsubPcs were grown by either train sublimation, slow vapour diffusion of heptane into toluene, slow evaporation of an organic solvent (*i.e.* acetone or methanol), two-solvent recrystallization or formed during rotary evaporation to remove solvent following purification by column chromatography. The influence of the axial group on the inter­molecular inter­actions, bowl depths and axial bond lengths was analysed and is discussed below.

### Inter­molecular inter­actions

The previously established method for describing the inter­molecular π–π inter­actions of BsubPcs uses some unique terminology for the side of the BsubPc bowl being considered and the alignment of iso­indole units between adjacent BsubPcs (Morse *et al.*, 2014 ▸; Virdo *et al.*, 2016 ▸; Claessens *et al.*, 2014 ▸; Farac *et al.*, 2024 ▸). First, the terms concave and convex are used to describe the side of the BsubPc bowl being considered, with concave being the ‘inside’ of the bowl and convex being the outer surface of the bowl [Fig. 1 ▸(*c*)]. Furthermore, the three outermost six-membered carbon rings of the BsubPc are called ‘heads’, while the six-membered rings containing the boron centre, one imine N atom and two pyrrole N atoms are called ‘tails’ [Fig. 1 ▸(*b*)] (Virdo *et al.*, 2016 ▸). With these terms, the inter­molecular π–π inter­actions of BsubPcs can be described as concave–concave, convex–convex, concave–convex, head-to-head, tail-to-tail and head-to-tail (Morse *et al.*, 2010 ▸, 2014 ▸). Inter­actions between the axial ligand and BsubPc π-system are also sometimes observed and are described as concave-to-axial ligand and convex-to-axial ligand (Morse *et al.*, 2010 ▸, 2014 ▸). Distances less than 4 Å are considered significant inter­molecular π–π inter­actions (Fulford *et al.*, 2012 ▸). All inter­actions involving a π-group are measured to the centroid of that group. Other inter­actions are considered significant if the inter­molecular distance between the two atoms is less than the sum of the van der Waals radii of the two atoms (Virdo *et al.*, 2013 ▸). For example, weak hy­dro­gen bonds with C—H⋯O, C—H⋯F and C—H⋯N contacts are characterized by H⋯O, H⋯F and H⋯N distances less than 2.72, 2.67 and 2.75 Å, respectively. Furthermore, C—H⋯halogen bonds are characterized by H⋯Cl and H⋯Br distances that are less than 2.95 and 3.05 Å, respectively.

#### Axially halogenated BsubPcs

**Figure 3:**
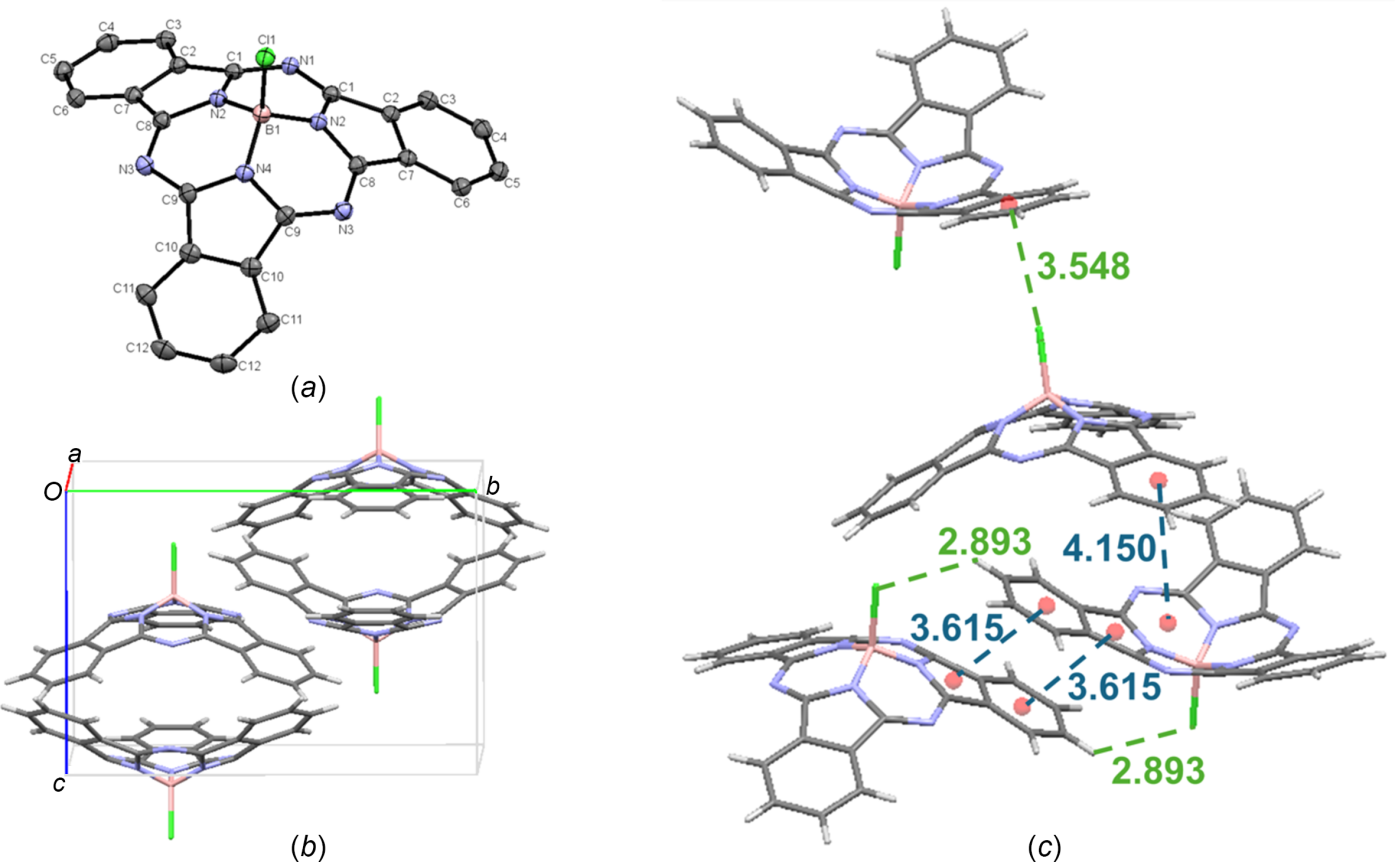
(*a*) The mol­ecular structure of Cl-BsubPc (**1**), with displacement ellipsoids drawn at the 50% probability level and H atoms removed for clarity, (*b*) the unit-cell packing and (*c*) significant inter­molecular inter­actions in the crystal structure. π–π inter­actions are shown in blue and all other inter­molecular inter­actions are shown in green. Atom colours: carbon – gray, hy­dro­gen – white, nitro­gen – blue, boron – light pink and chlorine – green.

The crystal structure of Cl-BsubPc [**1**; Fig. 3 ▸(*a*)] from train sublimation grown in this study shows good agreement with the previously reported structure (Virdo *et al.*, 2016 ▸). It arranges in the ortho­rhom­bic *Pnma* space group, with four mol­ecules in the unit cell [Fig. 3 ▸(*b*)] **[see Note 2]**. There are significant convex–convex head-to-head inter­actions, with a π_pyr_–π_Ph_ distance (pyr is pyrrole and Ph is phenyl) of 3.615 Å, and less significant concave–concave head-to-tail inter­actions, with an inter­molecular distance of 4.150 Å. There are also C—H⋯halogen inter­actions between the axial Cl and a π_Ph_ H atom of an adjacent mol­ecule (C—H_Ph_⋯Cl—B) at an inter­molecular distance of 2.893 Å. Upon extension of the unit cell, there are convex-to-axial ligand halogen–π (B—Cl⋯π_Ph_) inter­actions, with an inter­molecular distance of 3.548 Å. The inter­molecular inter­actions of Cl-BsubPc (**1**) are shown in Fig. 3 ▸(*c*).

**Figure 4:**
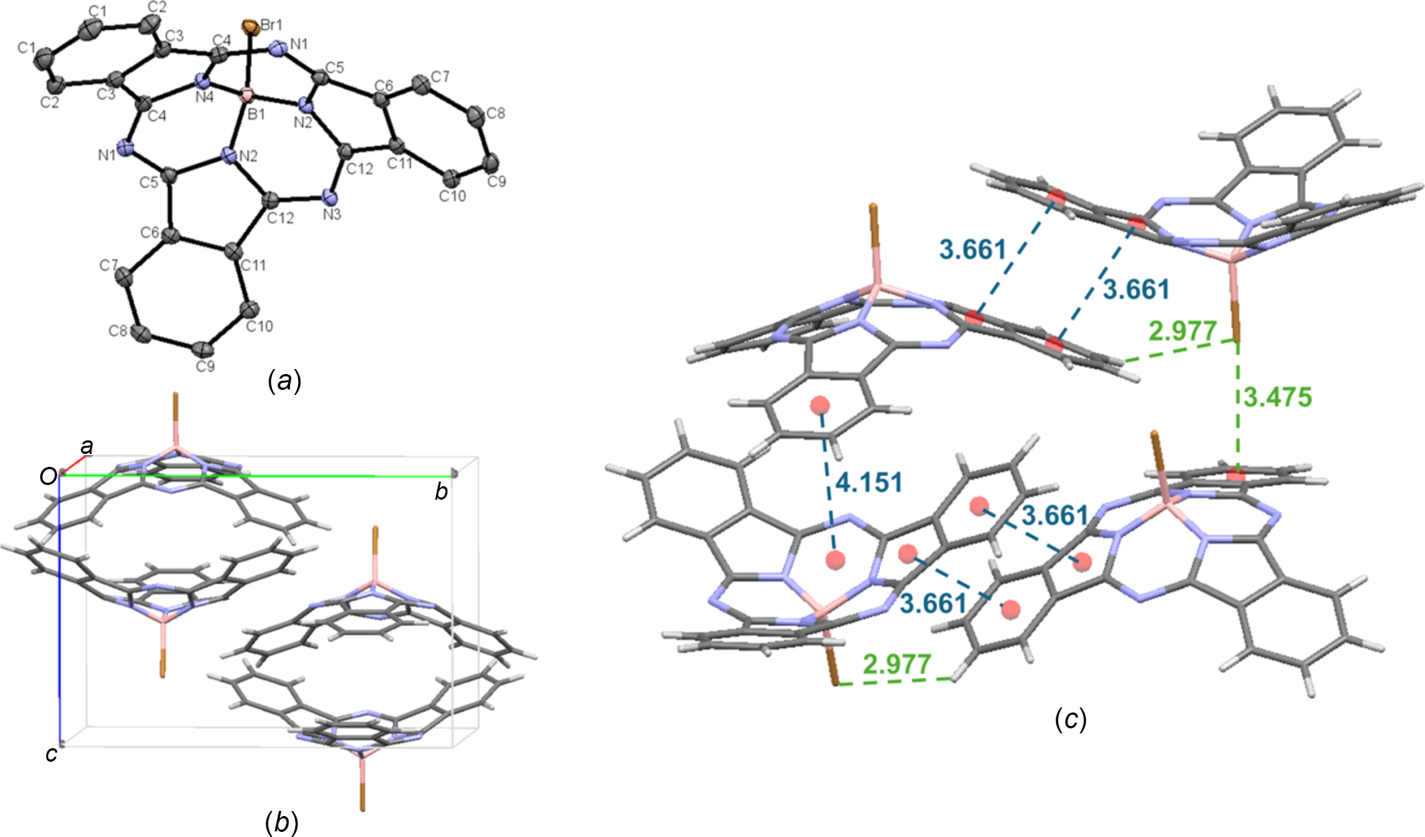
(*a*) The mol­ecular structure of Br-BsubPc (**2**), with displacement ellipsoids drawn at the 50% probability level and H atoms removed for clarity, (*b*) the unit-cell packing and (*c*) significant inter­molecular inter­actions in the crystal structure. π–π inter­actions are shown in blue and all other inter­molecular inter­actions are shown in green. Atom colours: carbon – gray, hy­dro­gen – white, nitro­gen – blue, boron – light pink and bromine – bronze.

It has been reported previously that a Br-BsubPc [**2**; Fig. 4 ▸(*a*)] crystal cannot be grown by train sublimation because the boron–bromine axial bond (B—Br) is not thermally stable at its sublimation temperature (Fulford *et al.*, 2012 ▸; Hildebrand *et al.*, 2022 ▸). However, within this study, we were able to obtain a sublimed crystal of Br-BsubPc during the attempted purification of TMSO-BsubPc (**13**) by train sublimation (350 °C), as there was some unreacted Br-BsubPc remaining in the crude product from the TMSO-BsubPc axial exchange reaction, and therefore was separated and crystallized during train sublimation. Like Cl-BsubPc, Br-BsubPc arranges in the ortho­rhom­bic *Pnma* space group, with four mol­ecules in the unit cell [Fig. 4 ▸(*b*)], which is consistent with the previously reported Br-BsubPc crystal structure using solvent-based crystal growth methods (Fulford *et al.*, 2012 ▸). The inter­molecular inter­actions observed in the Br-BsubPc crystal structure are very similar to those of the Cl-BsubPc crystal structure. Within the unit cell, Br-BsubPc displays concave–concave head-to-tail packing, with an inter­molecular distance of 4.151 Å, which is nearly identical to the distance observed for Cl-BsubPc. Upon extension outside the unit cell, more significant π–π inter­actions are seen, with convex–convex head-to-head inter­actions between adjacent mol­ecules at a π_pyr_–π_Ph_ distance of 3.661 Å. There are also C—H⋯halogen inter­actions between the axial Br atom and an adjacent π_Ph_ H atom (C—H_Ph_⋯Br—B), with an inter­molecular distance of 2.977 Å, which is slightly longer than what is seen in the Cl-BsubPc crystal structure due to the larger size of bromine com­pared to chlorine. Convex-to-axial ligand halogen–π (B—Br⋯π_Ph_) inter­actions are also observed, with an inter­molecular distance of 3.475 Å. This is shorter than the distance observed for the analogous inter­action for Cl-BsubPc. The inter­molecular inter­actions of Br-BsubPc (**2**) are shown in Fig. 4 ▸(*c*).

#### Axial boron–oxygen-bonded BsubPcs

**Figure 5:**
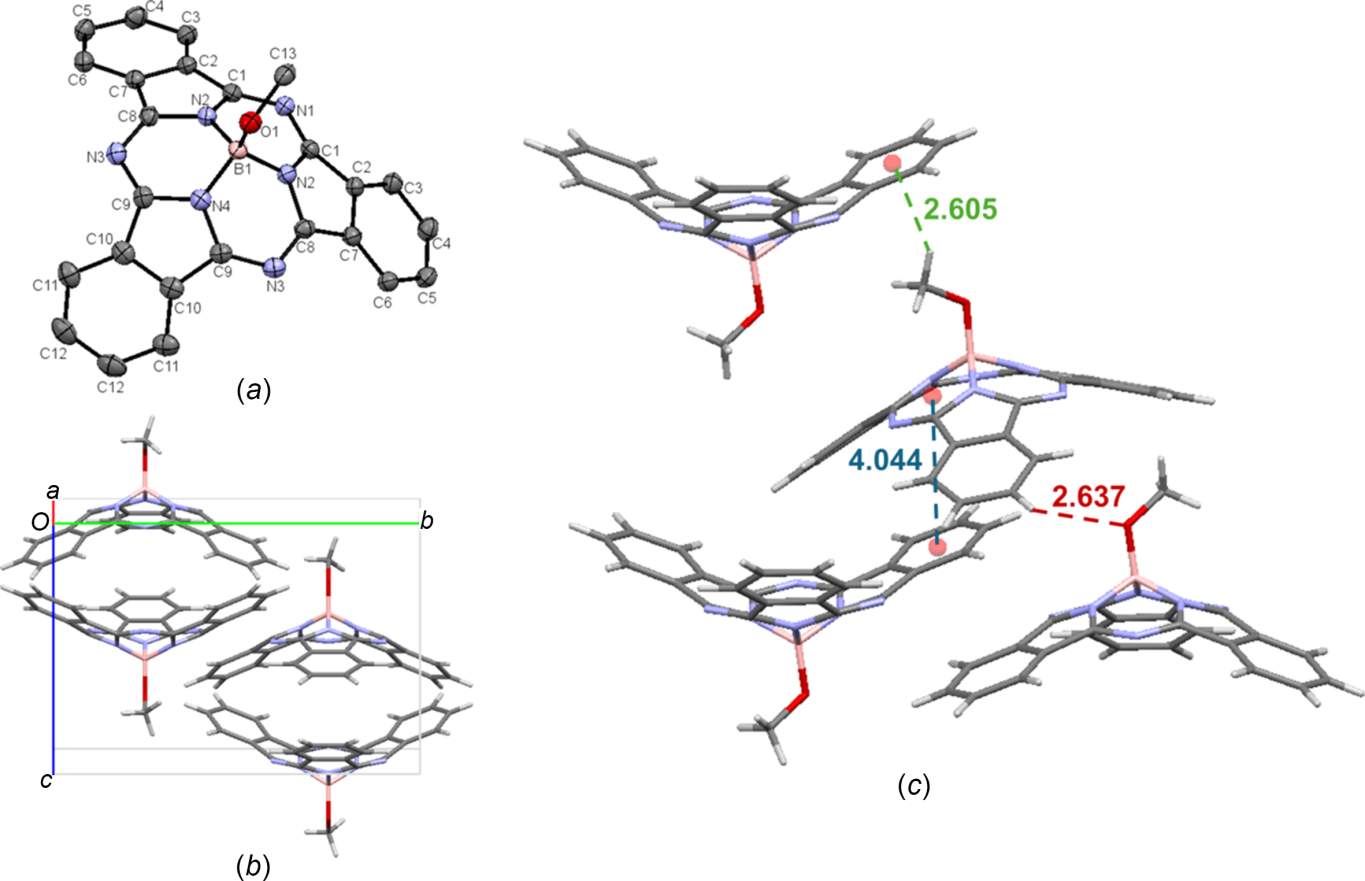
(*a*) The mol­ecular structure of MeO-BsubPc (**3**), with displacement ellipsoids drawn at the 50% probability level and H atoms removed for clarity, (*b*) the unit-cell packing and (*c*) significant inter­molecular inter­actions in the crystal structure. π–π inter­actions are shown in blue, weak hy­dro­gen bonds are shown in red and all other inter­molecular inter­actions are shown in green. Atom colours: carbon – gray, hy­dro­gen – white, nitro­gen – blue, boron – light pink and oxygen – red.

Crystals of MeO-BsubPc [**3**; Fig. 5 ▸(*a*)] were grown by train sublimation and arranged in the ortho­rhom­bic *Pnma* space group, with four mol­ecules in the unit cell [Fig. 5 ▸(*b*)]. The only π–π inter­actions are weak concave–concave head-to-tail inter­actions, with an inter­molecular distance of 4.044 Å. There is weak hy­dro­gen bonding between the axial O atom and an adjacent π_Ph_ H atom (C—H_Ph_⋯O—B), with an inter­molecular distance of 2.637 Å. Extending beyond the unit cell, there is a convex-to-axial ligand C—H_ax_⋯π_Ph_ inter­action, with an inter­molecular distance of 2.605 Å. The inter­molecular inter­actions of MeO-BsubPc (**3**) are shown in Fig. 5 ▸(*c*).

**Figure 6:**
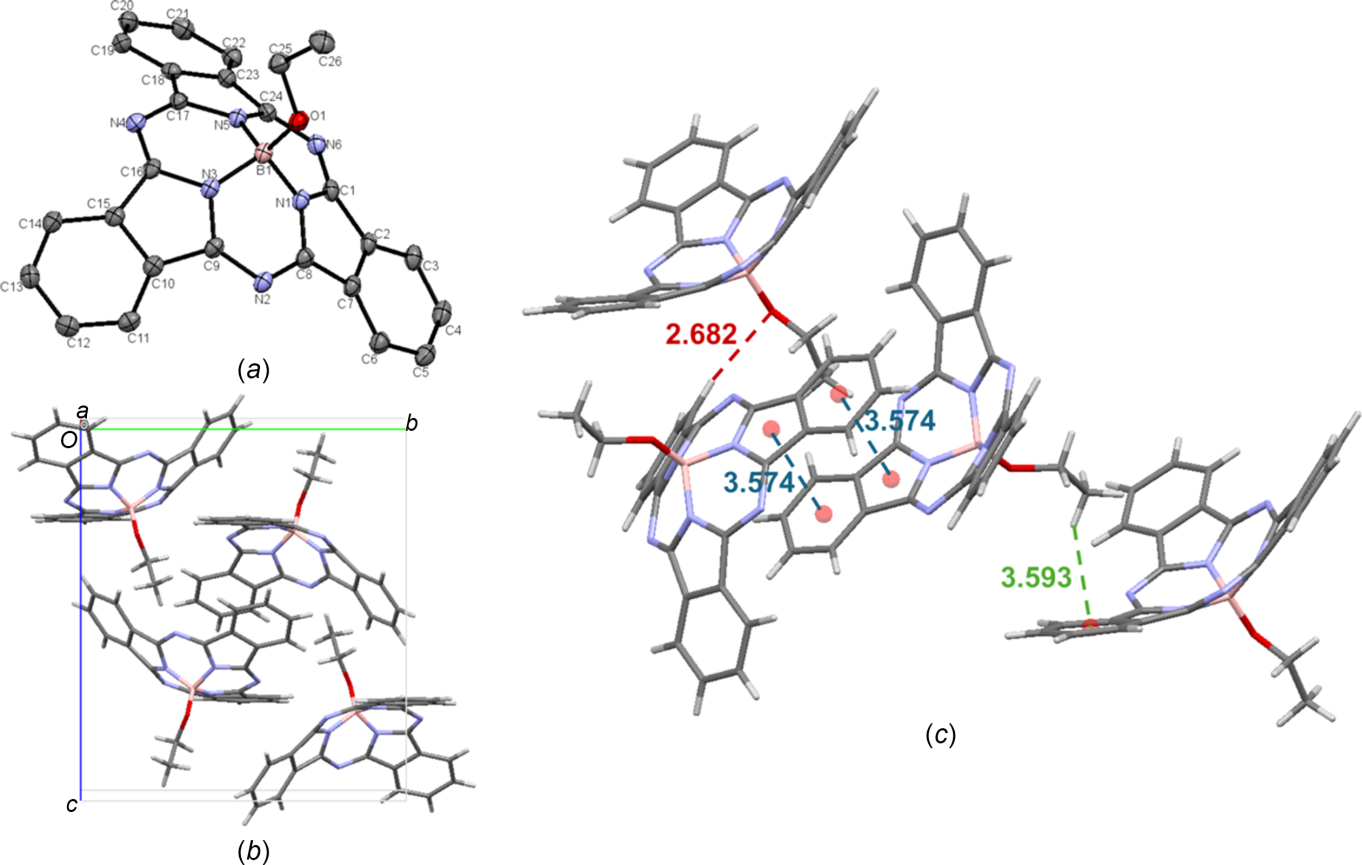
(*a*) The mol­ecular structure of EtO-BsubPc (**4-I**), with displacement ellipsoids drawn at the 50% probability level and H atoms removed for clarity, (*b*) the unit-cell packing and (*c*) significant inter­molecular inter­actions in the crystal structure. π–π inter­actions are shown in blue, weak hy­dro­gen bonds are shown in red and all other inter­actions are shown in green. Atom colours: carbon – gray, hy­dro­gen – white, nitro­gen – blue, boron – light pink and oxygen – red.

Crystals of EtO-BsubPc (**4**) were grown in two ways: train sublimation [**4-I**; Fig. 6 ▸(*a*)] and slow evaporation of acetone (**4-II**). The crystal structure was the same by both crystal growth methods. EtO-BsubPc is the only com­pound in the array that crystallizes in the monoclinic *P*2_1_/*n* space group. Starting with **4-I**, there are four mol­ecules in the unit cell [Fig. 6 ▸(*b*)] and the only π–π inter­actions are concave–concave head-to-head inter­actions, with a π_pyr_–π_Ph_ distance of 3.574 Å. There is weak hy­dro­gen bonding between the axial O atom and an adjacent π_Ph_ H atom (C—H_Ph_⋯O—B), with an inter­molecular distance of 2.682 Å, which is slightly longer than the analogous distance for MeO-BsubPc. Extending beyond the unit cell, there is a concave-to-axial ligand C_26_—H_ax_⋯π_Ph_ inter­action, with an inter­molecular distance of 3.593 Å. EtO-BsubPc is the only com­pound in the array with this type of inter­action. The inter­molecular inter­actions of EtO-BsubPc (**4-I**) are shown in Fig. 6 ▸(*c*).

**Figure 7:**
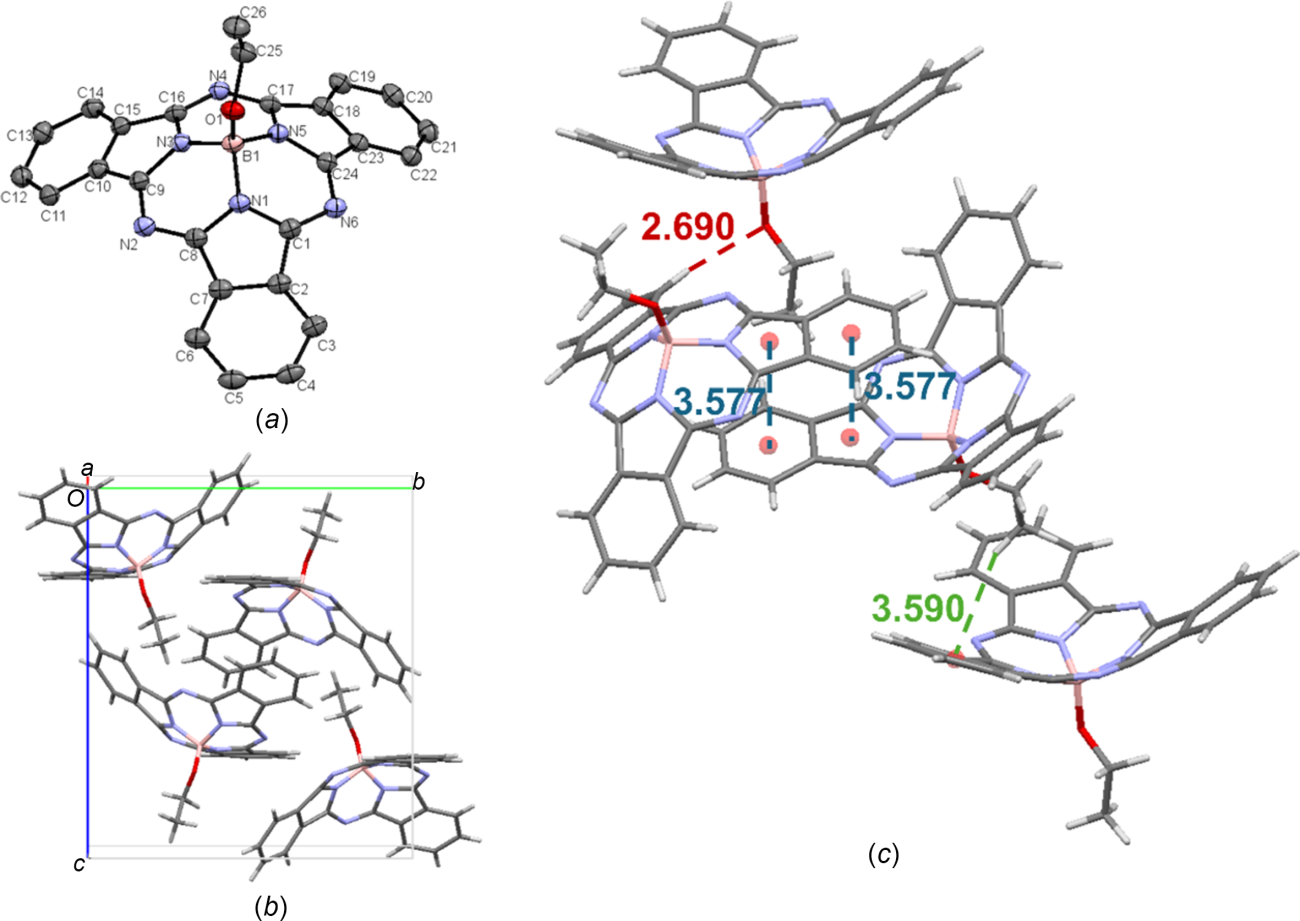
(*a*) The mol­ecular structure of EtO-BsubPc (**4-II**), with displacement ellipsoids drawn at the 50% probability level and H atoms removed for clarity, (*b*) the unit-cell packing and (*c*) significant inter­molecular inter­actions in the crystal structure. π–π inter­actions are shown in blue, weak hy­dro­gen bonds are shown in red and all other inter­actions are shown in green. Atom colours: carbon – gray, hy­dro­gen – white, nitro­gen – blue, boron – light pink and oxygen – red.

As mentioned above, the crystal structure of EtO-BsubPc from slow evaporation of acetone [**4-II**; Fig. 7 ▸(*a*)] was the same as the crystal structure from train sublimation (**4-I**), arranging in the monoclinic *P*2_1_/*n* space group, with four mol­ecules in the unit cell [Fig. 7 ▸(*b*)]. Identical inter­molecular inter­actions were seen for **4-I** and **4-II**, with very modest differences in the inter­molecular distances. The concave–concave head-to-head π_pyr_–π_Ph_ distance for **4-II** (3.577 Å) was slightly longer than for **4-I** (3.574 Å), whereas the concave-to-axial ligand C_26_—H_ax_⋯π_Ph_ inter­molecular distance for **4-II** (3.590 Å) was slightly shorter than for **4-I** (3.593 Å). Lastly, the weak hy­dro­gen bonding between the axial O atom and an adjacent π_Ph_ H atom (C—H_Ph_⋯O—B) for **4-II** (2.690 Å) was at a slightly longer inter­molecular distance than for **4-I** (2.682 Å). The inter­molecular inter­actions of EtO-BsubPc (**4-II**) are shown in Fig. 7 ▸(*c*).

**Figure 8:**
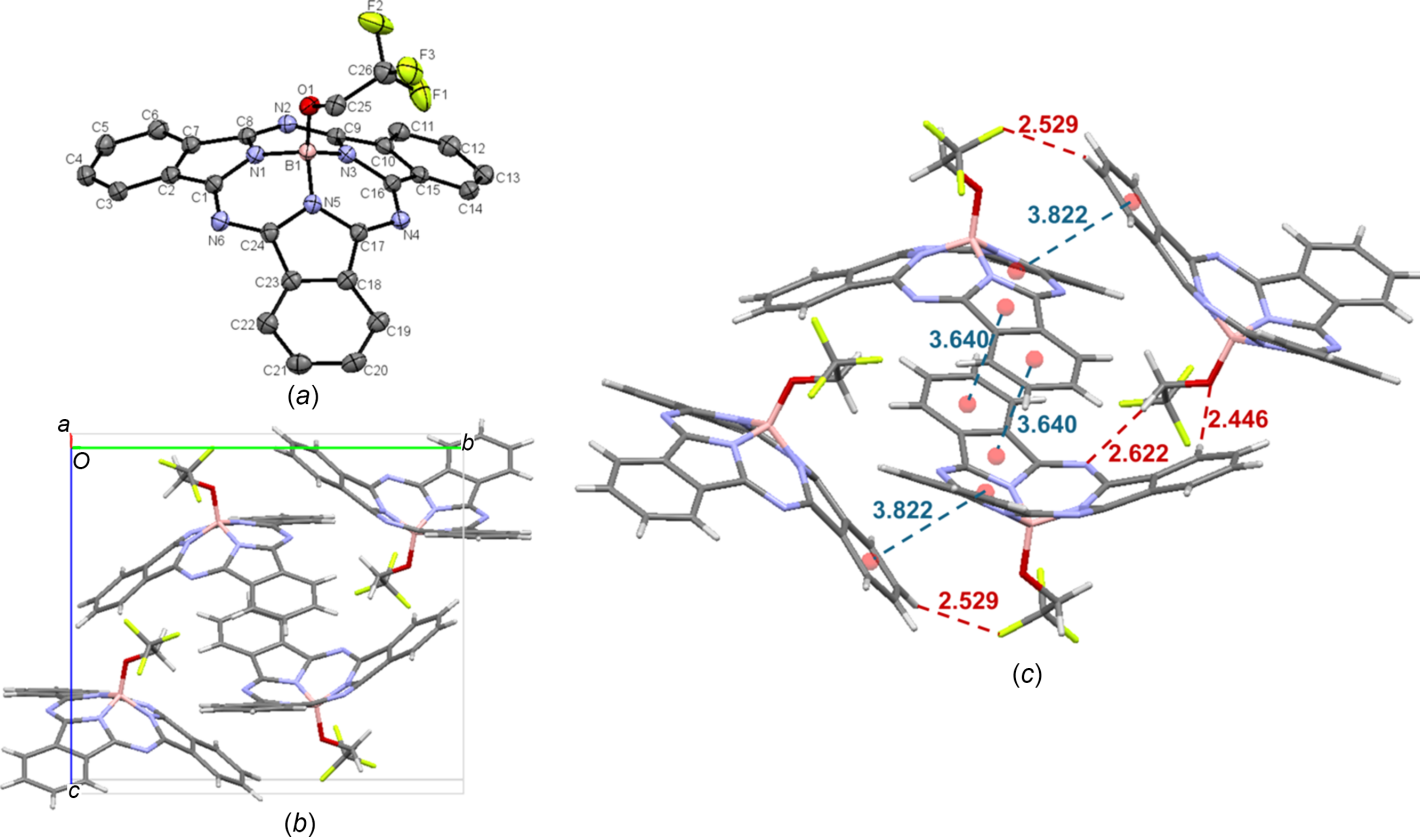
(*a*) The mol­ecular structure of F_3_EtO-BsubPc (**5**), with displacement ellipsoids drawn at the 50% probability level and H atoms removed for clarity, (*b*) the unit-cell packing and (*c*) significant inter­molecular inter­actions in the crystal structure. π–π inter­actions are shown in blue and weak hy­dro­gen bonds are shown in red. Atom colours: carbon – gray, hy­dro­gen – white, nitro­gen – blue, boron – light pink, oxygen – red and fluorine – green.

F_3_EtO-BsubPc [**5**; Fig. 8 ▸(*a*)] crystals were grown by train sublimation, organizing in the monoclinic *P*2_1_/*c* space group, with four mol­ecules in the unit cell [Fig. 8 ▸(*b*)]. Similar to EtO-BsubPc, the most significant π–π inter­actions are concave–concave head-to-head inter­actions, although the π_pyr_–π_Ph_ distance is slightly longer at 3.640 Å. Unlike EtO-BsubPc, convex–convex head-to-tail inter­actions are observed, with an inter­molecular distance of 3.822 Å. The axial group also has significant inter­actions, with weak hy­dro­gen bonding between the axial O atom and an adjacent π_Ph_ H atom (C—H_Ph_⋯O—B = 2.446 Å), an axial F atom with an adjacent π_Ph_ H atom (C—H_Ph_⋯F—C = 2.529 Å) and an axial methyl­ene H atom with an adjacent imine N atom (C_25_—H_ax_⋯N_imine_ = 2.622 Å). F_3_EtO-BsubPc is the only alk­oxy derivative that does not contain any concave- or convex-to-axial ligand inter­actions, indicating that when H atoms are replaced with F atoms, weak hy­dro­gen bonding dominates over axial–π inter­actions. The inter­molecular inter­actions of F_3_EtO-BsubPc (**5**) are shown in Fig. 8 ▸(*c*).

**Figure 9:**
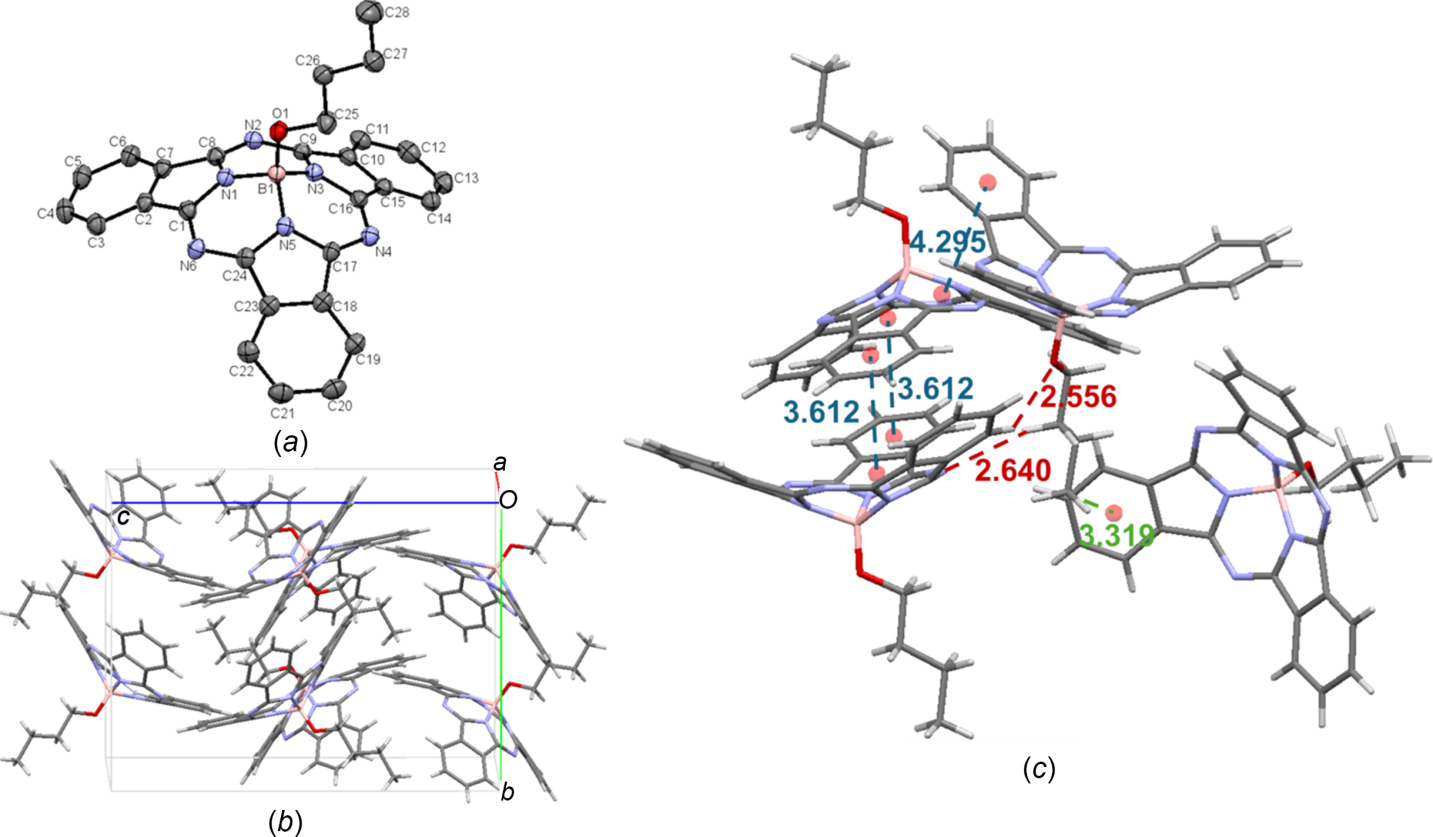
(*a*) The mol­ecular structure of ButO-BsubPc (**6**), with displacement ellipsoids drawn at the 50% probability level and H atoms removed for clarity, (*b*) the unit-cell packing and (*c*) significant inter­molecular inter­actions in the crystal structure. π–π inter­actions are shown in blue, weak hy­dro­gen bonds are shown in red and all other inter­molecular inter­actions are shown in green. Atom colours: carbon – gray, hy­dro­gen – white, nitro­gen – blue, boron – light pink and oxygen – red.

ButO-BsubPc [**6**; Fig. 9 ▸(*a*)] is the only com­pound in the array to crystallize in the ortho­rhom­bic *Pbca* space group, with eight mol­ecules in the unit cell [Fig. 9 ▸(*b*)]. There are significant concave–concave head-to-head inter­actions, with a π_pyr_–π_Ph_ distance of 3.612 Å, and less significant convex–convex head-to-tail inter­actions, with an inter­molecular distance of 4.295 Å. There are also significant inter­actions involving the axial group, including weak hy­dro­gen bonding between the axial O atom and a neighbouring π_Ph_ H atom (C—H_Ph_⋯O—B = 2.556 Å), and between an axial methyl­ene H atom and an adjacent imine N atom (C_26_—H_ax_⋯N_imine_ = 2.640 Å). There is also a convex-to-axial ligand C_28_—H_ax_⋯π_Ph_ inter­action between an axial methyl H atom and the π_Ph_ group of a neighbouring mol­ecule (3.319 Å). The inter­molecular inter­actions of ButO-BsubPc (**6**) are shown in Fig. 9 ▸(*c*).

**Figure 10:**
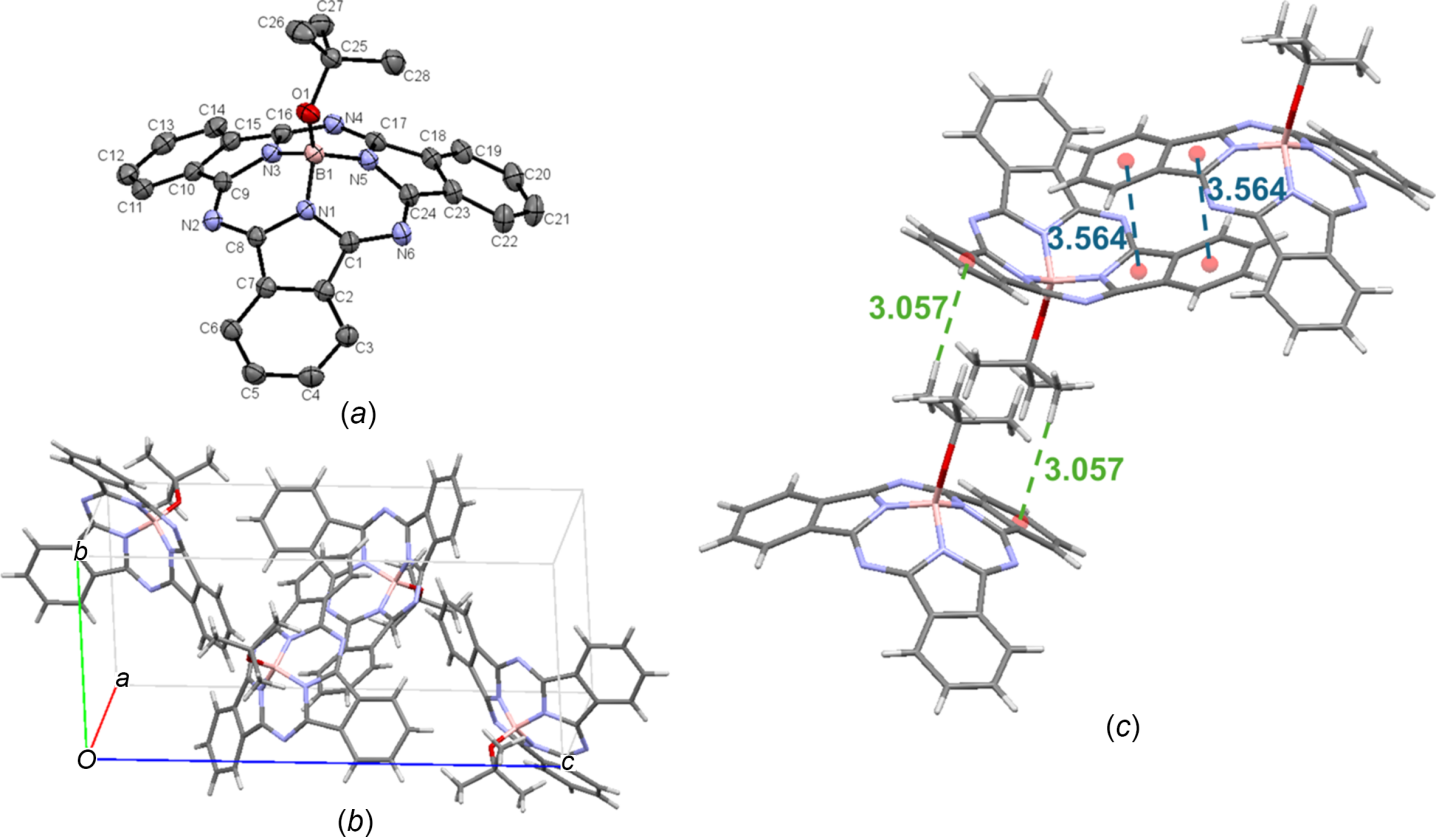
(*a*) The mol­ecular structure of tButO-BsubPc (**7**), with displacement ellipsoids drawn at the 50% probability level and H atoms removed for clarity, (*b*) the unit-cell packing and (*c*) significant inter­molecular inter­actions in the crystal structure. π–π inter­actions are shown in blue and all other inter­molecular inter­actions are shown in green. Atom colours: carbon – gray, hy­dro­gen – white, nitro­gen – blue, boron – light pink and oxygen – red.

tButO-BsubPc [**7**; Fig. 10 ▸(*a*)] arranges in the monoclinic *P*2_1_/*c* space group, with four mol­ecules in the unit cell [Fig. 10 ▸(*b*)]. Within the unit cell, the only significant π–π inter­action is a concave–concave head-to-head inter­action, with a π_pyr_–π_Ph_ distance of 3.564 Å, which is shorter than the distance observed for EtO-BsubPc (**4**), F_3_EtO-BsubPc (**5**) and ButO-BsubPc (**6**). Upon extension of the unit cell, there is a convex-to-axial ligand C_27_—H_ax_⋯π_Ph_ inter­action between an axial H atom and an adjacent π_Ph_ group (3.057 Å). No hy­dro­gen bonding is observed for tButO-BsubPc. The inter­molecular inter­actions of tButO-BsubPc (**7**) are shown in Fig. 10 ▸(*c*).

**Figure 11:**
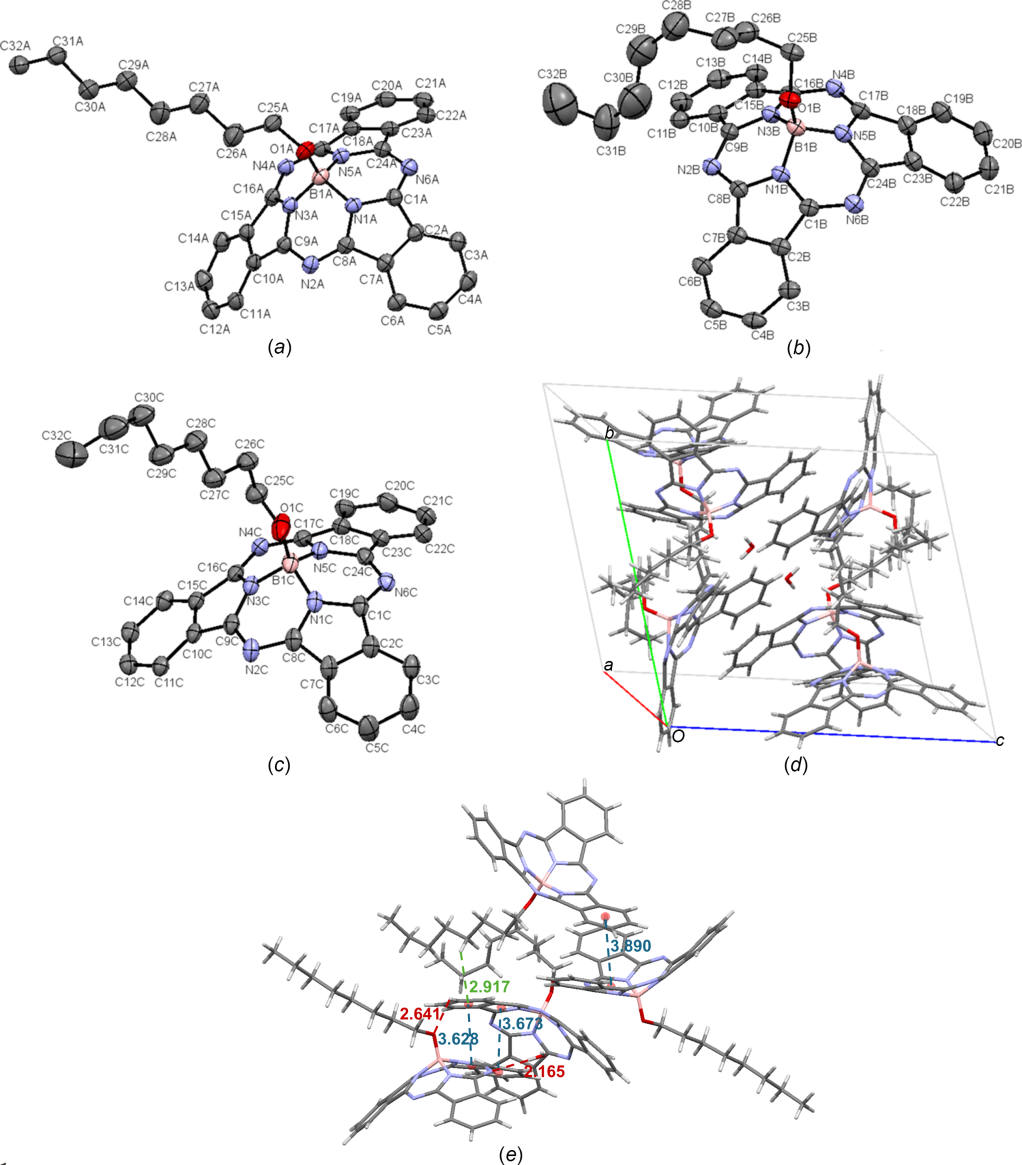
The mol­ecular structures of (*a*) OctO-BsubPc mol­ecule *A* (**8-A**), (*b*) OctO-BsubPc mol­ecule *B* (**8-B**) and (*c*) OctO-BsubPc mol­ecule *C* (**8-C**), with displacement ellipsoids drawn at the 50% probability and H atoms removed for clarity. (*d*) The unit-cell packing of **8** and (*e*) significant inter­molecular inter­actions in the crystal structure of **8**. π–π inter­actions are shown in blue, strong and weak hy­dro­gen bonds are shown in red, and all other inter­molecular inter­actions are shown in green. Atom colours: carbon – gray, hy­dro­gen – white, nitro­gen – blue, boron – light pink and oxygen – red.

A diffraction-quality crystal of OctO-BsubPc (**8**) was difficult to obtain, likely due to the length of the axial alkyl chain preventing close packing of neighbouring mol­ecules. After several unsuccessful attempts to grow crystals by train sublimation and solvent-based methods, such as slow evaporation and slow vapour diffusion, diffraction-quality crystals of OctO-BsubPc were successfully grown by slow evaporation of methanol, arranging in the triclinic *P*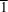 space group. There are three independent OctO-BsubPc mol­ecules in the asymmetric unit, which are referred to as **8-A** [Fig. 11 ▸(*a*)], **8-B** [Fig. 11 ▸(*b*)] and **8-C** [Fig. 11 ▸(*c*)], and 0.5 mol­ecules of water for every three mol­ecules of OctO-BsubPc [Fig. 11 ▸(*d*)]. **8-B** and **8-C** have disorder in the octyl chains. Within the unit cell, there appears to be concave–convex head-to-head packing between **8-A** and **8-B**, although the alignment is not perfect. The two π_pyr_–π_Ph_ distances between adjacent mol­ecules are not the same, at 3.628 and 3.673 Å. There also appears to be a concave–convex head-to-tail inter­action between **8-A** and **8-C**, with an inter­molecular distance of 3.890 Å, but again, the alignment is not perfect between the two adjacent mol­ecules. The length of the axial octyl chain is likely responsible for the lack of perfect alignment. There is weak hy­dro­gen bond between the axial O atom of **8-A** and a π_Ph_ H atom of the adjacent **8-B** mol­ecule (C—H_Ph_⋯O—B), with an inter­molecular distance of 2.641 Å. There is also hy­dro­gen bonding between a water mol­ecule and the imine N atom of an adjacent **8-A** mol­ecule (2.165 Å). Lastly, convex-to-axial ligand C—H⋯π inter­actions are observed between an axial H atom of **8-C** and the π_Ph_ group of **8-B**, with a C—H_ax_⋯π_Ph_ distance of 2.971 Å. The inter­molecular inter­actions of OctO-BsubPc (**8**) are shown in Fig. 11 ▸(*e*).

**Figure 12:**
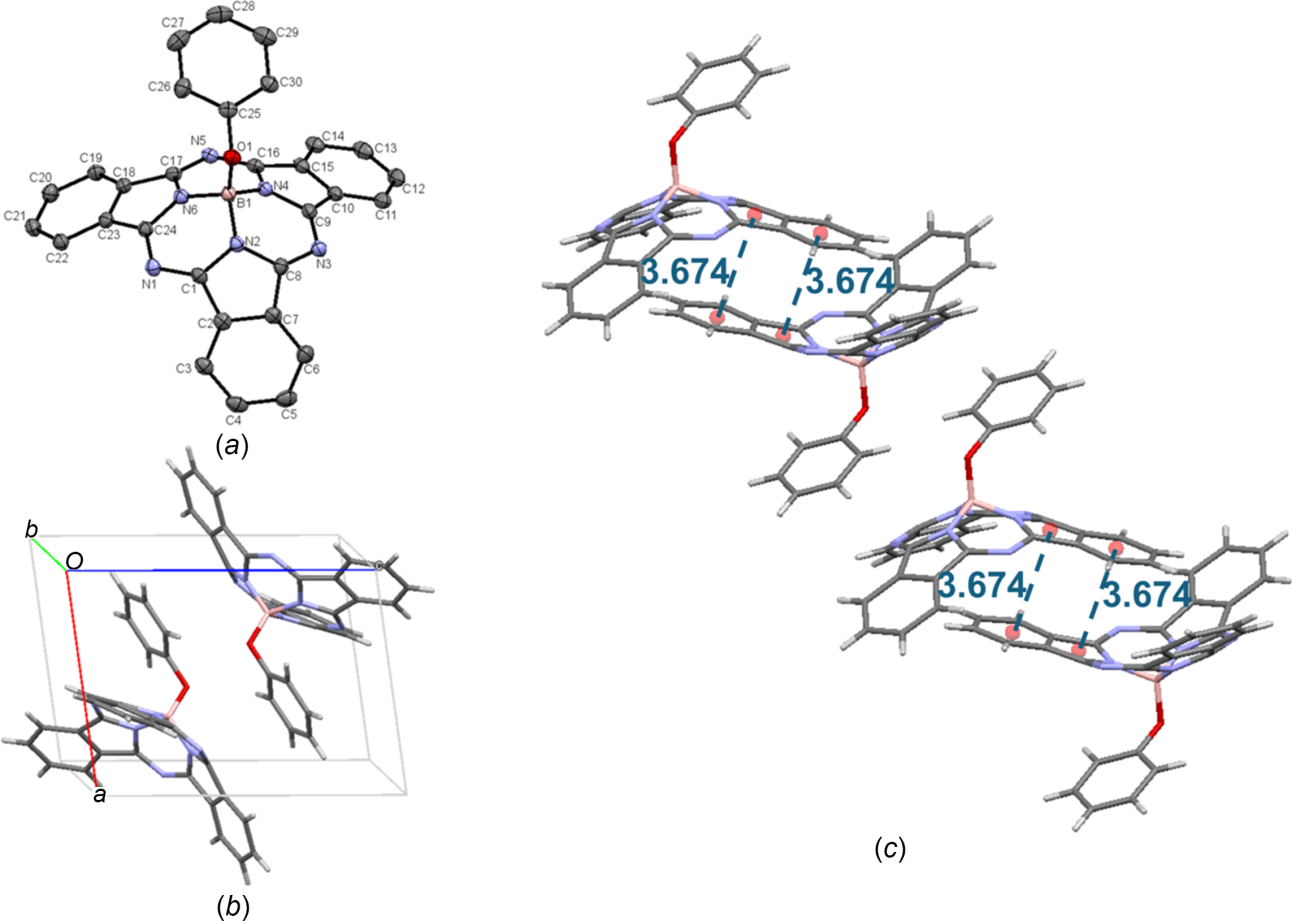
(*a*) The mol­ecular structure of PhO-BsubPc (**9**), with displacement ellipsoids drawn at the 50% probability level and H atoms removed for clarity, (*b*) the unit-cell packing and (*c*) significant inter­molecular inter­actions in the crystal structure. π–π inter­actions are shown in blue. Atom colours: carbon – gray, hy­dro­gen – white, nitro­gen – blue, boron – light pink and oxygen – red.

Crystals of PhO-BsubPc [**9**; Fig. 12 ▸(*a*)] were grown by slow vapour diffusion of heptane into toluene, arranging in the triclinic *P*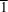 space group, with two mol­ecules in the unit cell [Fig. 12 ▸(*b*)], as was reported previously (Paton *et al.*, 2011 ▸). While there are no π–π inter­actions within the unit cell, upon extension, concave–concave head-to-head packing is ob­ser­ved, with a π_pyr_–π_Ph_ distance of 3.674 Å. There do not appear to be any π–π inter­actions between the axial group and the iso­indole groups of adjacent mol­ecules, or any other inter­actions involving the axial group. The inter­molecular inter­actions of PhO-BsubPc (**9**) are shown in Fig. 12 ▸(*c*).

**Figure 13:**
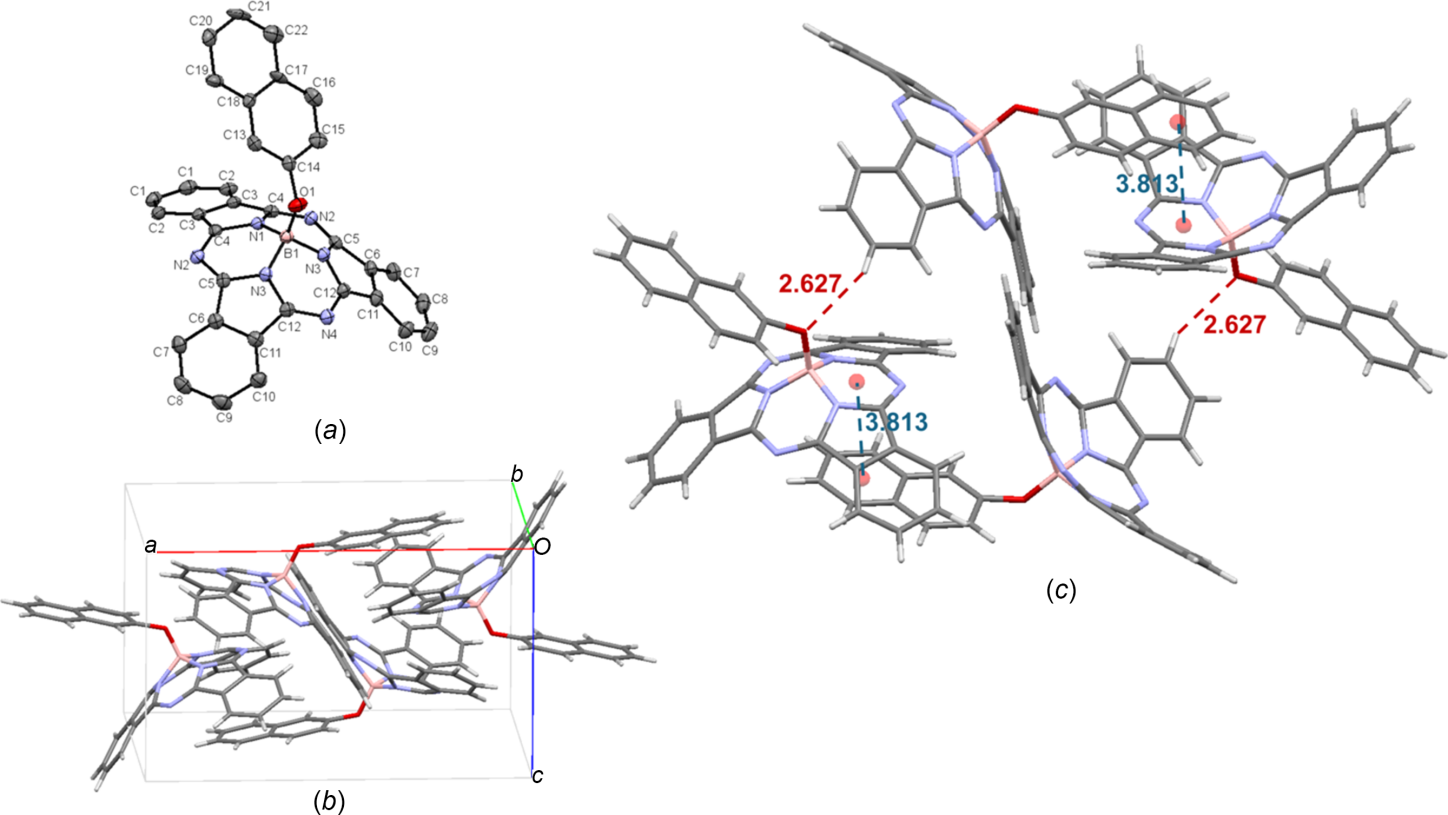
(*a*) The mol­ecular structure of naphth­oxy-BsubPc (**10**), with displacement ellipsoids drawn at the 50% probability level and H atoms removed for clarity, (*b*) the unit-cell packing and (*c*) significant inter­molecular inter­actions in the crystal structure. π–π inter­actions are shown in blue and weak hy­dro­gen bonds are shown in red. Atom colours: carbon – gray, hy­dro­gen – white, nitro­gen – blue, boron – light pink and oxygen – red.

Crystals of naphth­oxy-BsubPc [**10**; Fig. 13 ▸(*a*)] were grown by train sublimation and the resulting crystal structure is identical to a previous report from our group (Paton *et al.*, 2013 ▸). Naphth­oxy-BsubPc arranges in the ortho­rhom­bic *Pnma* space group, with four mol­ecules in the unit cell [Fig. 13 ▸(*b*)]. The naphth­oxy axial group causes a disruption in the π–π inter­actions of the iso­indole units of neighbouring BsubPcs, instead containing concave-to-axial ligand π–π inter­actions between the centroid of the outer ring of the naphth­oxy unit and the tail of the BsubPc, with an inter­molecular distance of 3.813 Å. There is also weak hy­dro­gen bonding between the axial O atom and a neighbouring π_Ph_ H atom (2.627 Å). The inter­molecular inter­actions of naphth­oxy-BsubPc (**10**) are shown in Fig. 13 ▸(*c*).

**Figure 14:**
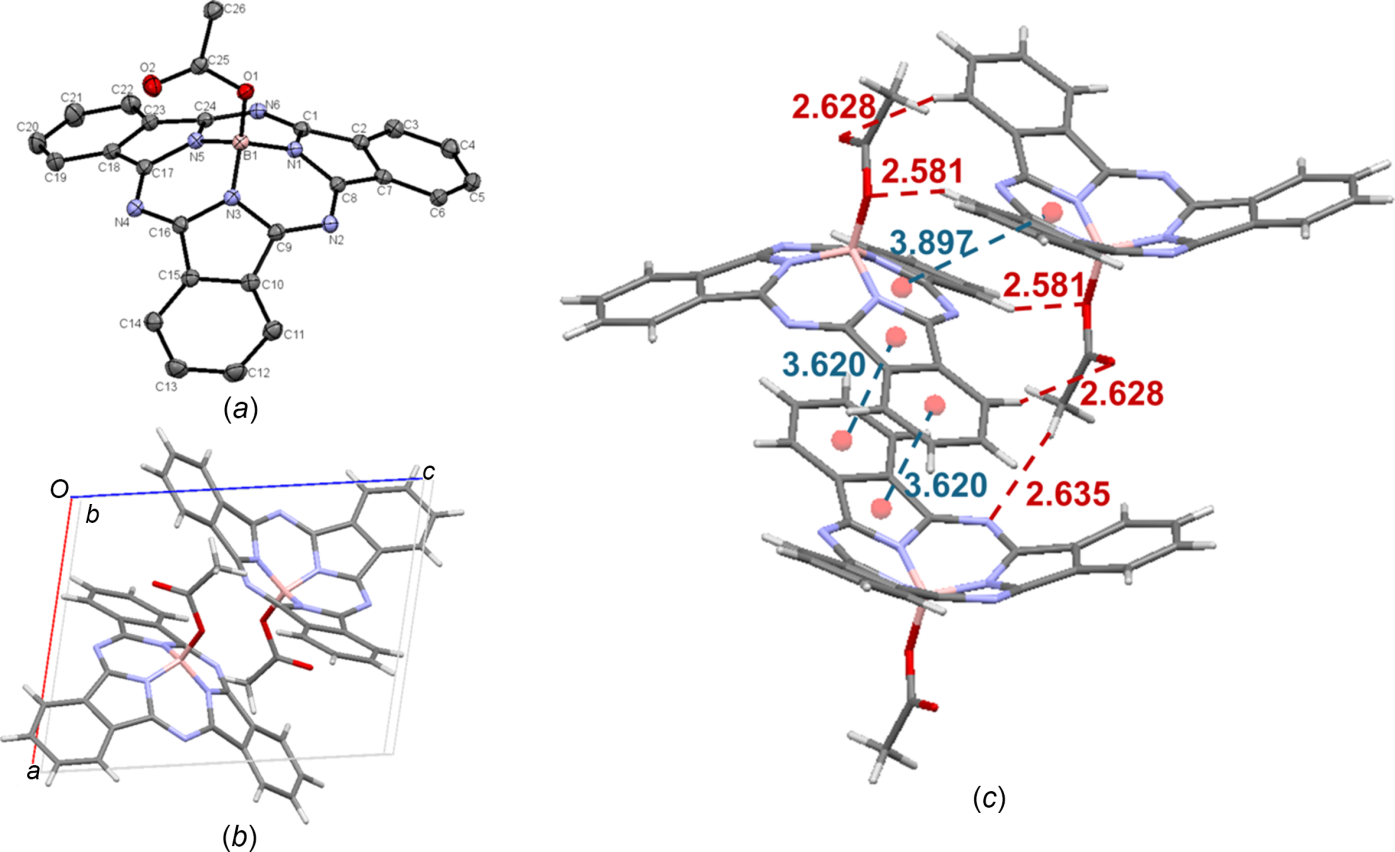
(*a*) The mol­ecular structure of acetate-BsubPc (**11**), with displacement ellipsoids drawn at the 50% probability level and H atoms removed for clarity, (*b*) the unit-cell packing and (*c*) significant inter­molecular inter­actions in the crystal structure. π–π inter­actions are shown in blue and weak hy­dro­gen bonds are shown in red. Atom colours: carbon – gray, hy­dro­gen – white, nitro­gen – blue, boron – light pink and oxygen – red.

Moving on to the carboxyl­ate derivatives (**11** and **12**), crystals of acetate-BsubPc [**11**; Fig. 14 ▸(*a*)] were grown by train sublimation, showing good agreement with the previously reported structure from slow vapour diffusion of heptane into toluene (Lessard & Bender, 2013 ▸). Acetate-BsubPc crystallizes in the triclinic *P*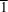 space group, with two mol­ecules in the unit cell [Fig. 14 ▸(*b*)]. Packing within the unit cell appears to be largely dictated by the axial-group inter­actions, with weak hy­dro­gen bonding between the axial O atom and an adjacent π_Ph_ H atom (C—H_Ph_⋯O—B = 2.581 Å), the axial carbonyl O atom and an adjacent π_Ph_ H atom (C—H_Ph_⋯O=C = 2.628 Å), and an axial H atom and an adjacent imine N atom (C—H_ax_⋯N_imine_ = 2.635 Å). The only π–π inter­actions ob­served in the unit cell are convex–convex tail-to-tail inter­actions, with an inter­molecular distance of 3.897 Å. Looking beyond the unit cell, there are concave–concave head-to-head inter­actions, with a π_pyr_–π_Ph_ distance of 3.620 Å. The inter­molecular inter­actions of acetate-BsubPc (**11**) are shown in Fig. 14 ▸(*c*).

**Figure 15:**
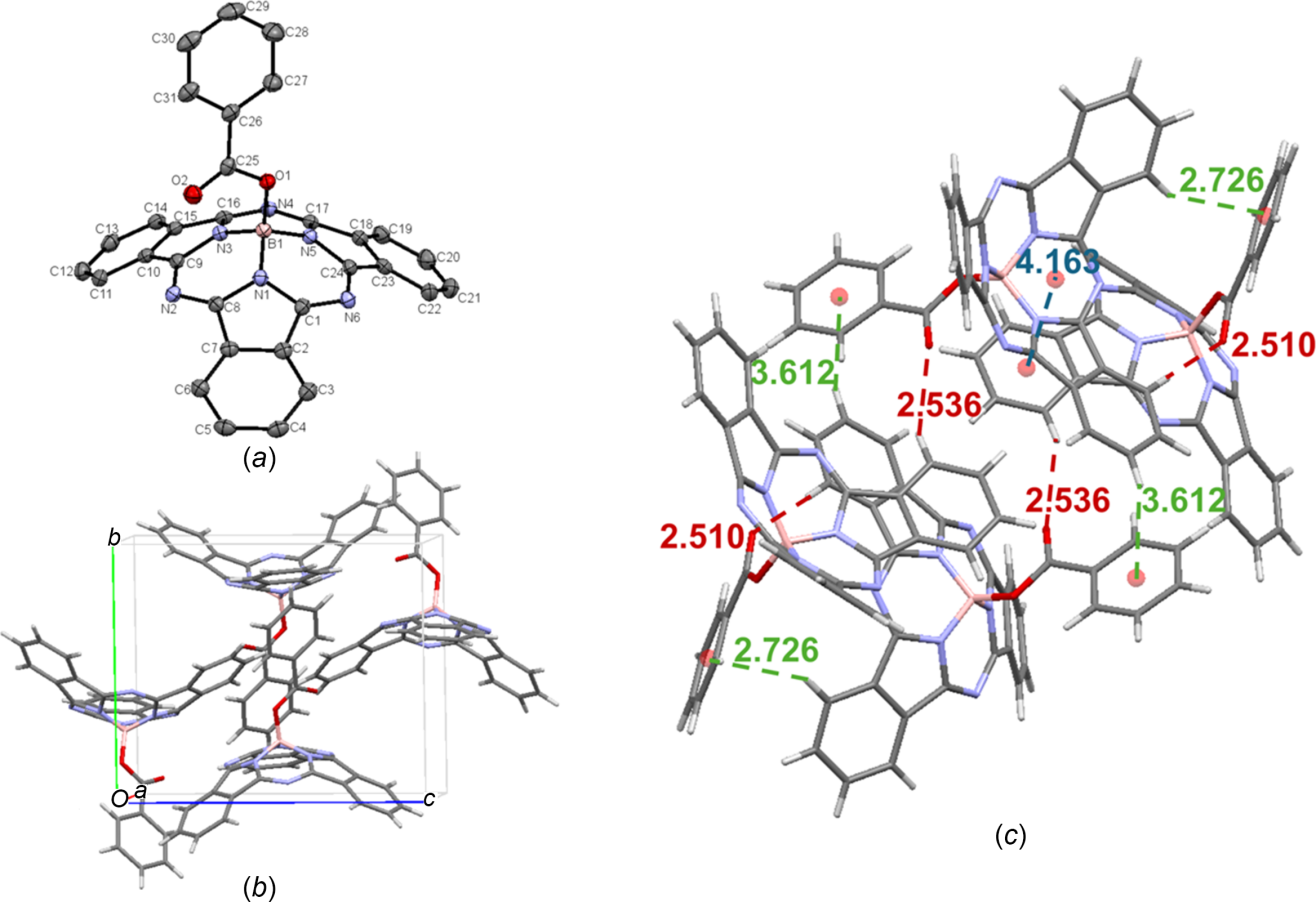
(*a*) The mol­ecular structure of benzoate-BsubPc (**12**), with displacement ellipsoids drawn at the 50% probability level and H atoms removed for clarity, (*b*) the unit-cell packing and (*c*) significant inter­molecular inter­actions in the crystal structure. π–π inter­actions are shown in blue, weak hy­dro­gen bonds are shown in red and all other inter­molecular inter­actions are shown in green. Atom colours: carbon – gray, hy­dro­gen – white, nitro­gen – blue, boron – light pink and oxygen – red.

Crystals of benzoate-BsubPc [**12**; Fig. 15 ▸(*a*)] were grown by train sublimation, again showing good agreement with the previously reported structure from slow vapour diffusion of heptane into toluene (Lessard & Bender, 2013 ▸). Benzoate-BsubPc arranges in the monoclinic *P*2_1_/*c* space group, with four mol­ecules in the unit cell [Fig. 15 ▸(*b*)], which is different from acetate-BsubPc. The benzoate axial group seems to disrupt the BsubPc π–π inter­actions, as only weak convex–convex head-to-tail inter­actions are observed, with an inter­molecular distance of 4.163 Å. As with acetate-BsubPc, the most significant inter­molecular inter­actions for benzoate-BsubPc involve the axial group. First, C—H_Ph_⋯π_ax_ inter­actions are observed, with the closest inter­molecular distances being 2.726 or 3.612 Å, depending on which mol­ecules in the unit cell are being considered. Weak hy­dro­gen bonding between the axial carbonyl O atom and adjacent π_Ph_ H atoms (C—H_Ph_⋯O=C) is also observed, with inter­molecular distances of 2.510 or 2.536 Å, depending on the mol­ecules in the unit cell being considered. The inter­molecular inter­actions of benzoate-BsubPc (**12**) are shown in Fig. 15 ▸(*c*).

**Figure 16:**
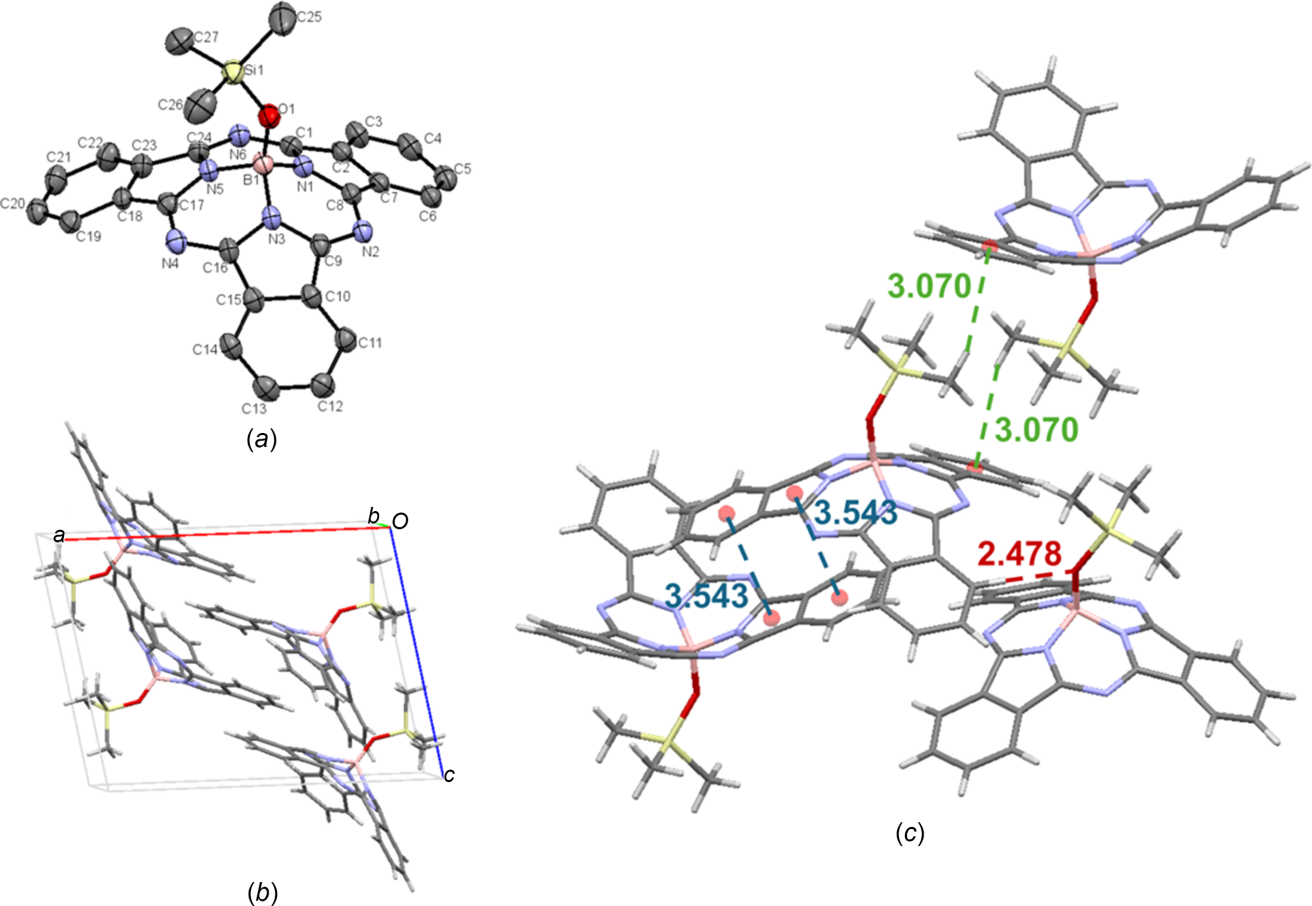
(*a*) The mol­ecular structure of TMSO-BsubPc (**13**), with displacement ellipsoids drawn at the 50% probability level and H atoms removed for clarity, (*b*) the unit-cell packing and (*c*) significant inter­molecular inter­actions in the crystal structure. π–π inter­actions are shown in blue, weak hy­dro­gen bonds are shown in red and all other inter­molecular inter­actions are shown in green. Atom colours: carbon – gray, hy­dro­gen – white, nitro­gen – blue, boron – light pink, oxygen – red and silicon – yellow.

The packing of the crystal of TMSO-BsubPc [**13**; Fig. 16 ▸(*a*)] is analogous to that of tButO-BsubPc (**7**), with the only structural difference between the two com­pounds being the replacement of the quaternary C atom of the axial *tert*-butyl group in tButO-BsubPc with an Si atom in TMSO-BsubPc. The crystals of both com­pounds were grown serendipitously during rotary evaporation of DCM/hexa­nes following purification by column chromatography. Both arrange in the monoclinic *P*2_1_/*c* space group, with four mol­ecules in the unit cell [Fig. 16 ▸(*b*)], and display concave–concave head-to-head packing. The inter­molecular π_pyr_–π_Ph_ distance for TMSO-BsubPc (3.543 Å) is slightly shorter than for tButO-BsubPc, while the convex-to-axial ligand C_26_—H_ax_⋯π_Ph_ inter­action has a slightly longer inter­molecular distance (3.070 Å) for TMSO-BsubPc than for tButO-BsubPc. There is also weak hy­dro­gen bonding observed between an axial H atom and an adjacent π_Ph_ H atom (C—H_Ph_⋯O—B = 2.478 Å), which is not observed for tButO-BsubPc. This may result from the longer bond length between oxygen and silicon (1.638 Å) in TMSO-BsubPc com­pared to oxygen and carbon (1.440 Å) in tButO-BsubPc, allowing for closer inter­actions with the axial O atom due to less steric hindrance directly around the O atom. The inter­molecular inter­actions of TMSO-BsubPc (**13**) are shown in Fig. 16 ▸(*c*).

#### Axial boron–sulfur- and boron–nitro­gen-bonded BsubPcs

**Figure 17:**
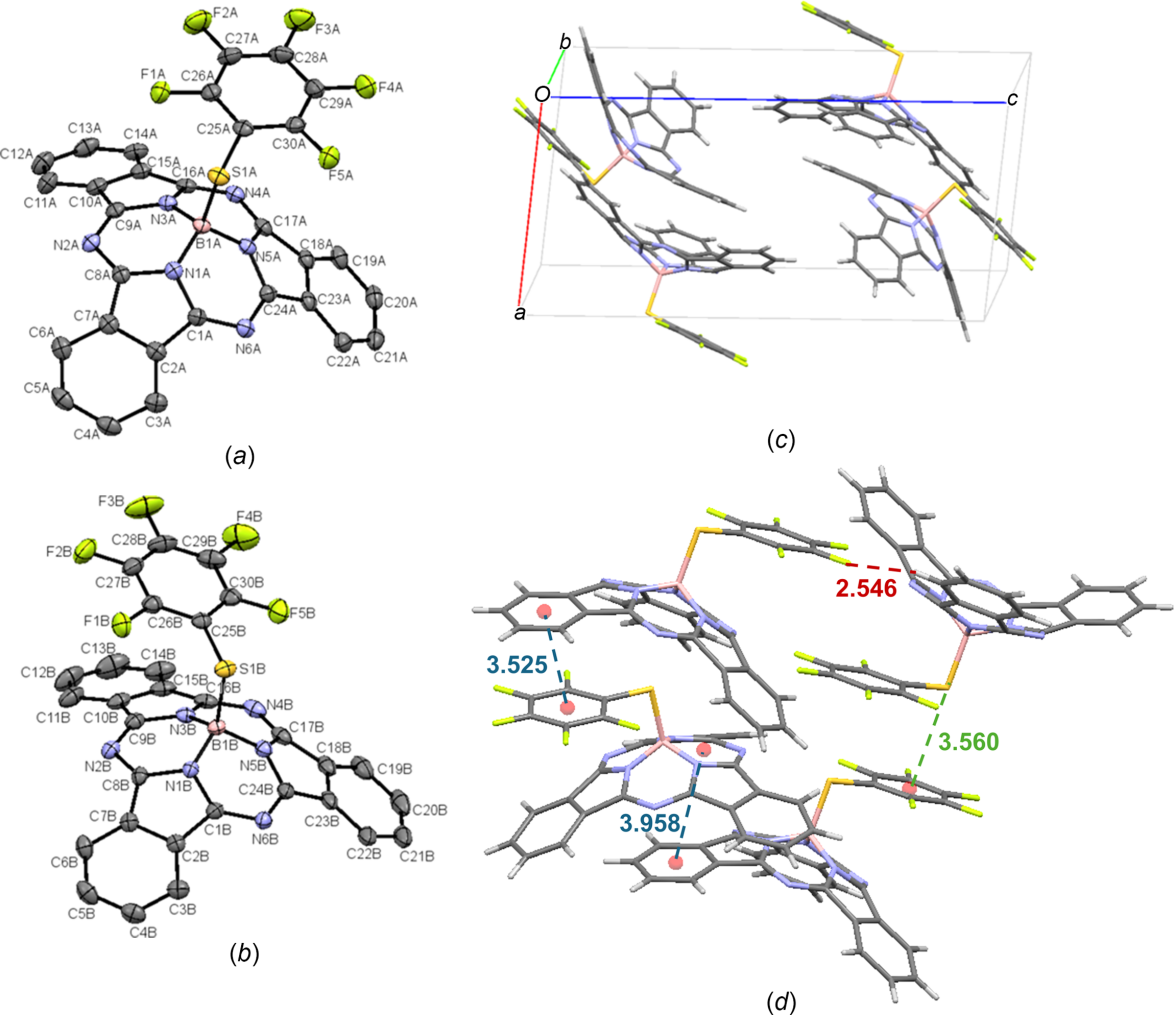
The mol­ecular structures of (*a*) F_5_PhS-BsubPc mol­ecule *A* (**14-A**) and (*b*) F_5_PhS-BsubPc mol­ecule *B* (**14-B**), with displacement ellipsoids drawn at the 50% probability level and H atoms removed for clarity. (*c*) The unit-cell packing and (*d*) significant inter­molecular inter­actions in the crystal structure of **14**. π–π inter­actions are shown in blue, weak hy­dro­gen bonds are shown in red and all other inter­molecular inter­actions are shown in green. Atom colours: carbon – gray, hy­dro­gen – white, nitro­gen – blue, boron – light pink, sulfur – orange and fluorine – green.

Crystals of F_5_PhS-BsubPc (**14**) were grown by slow vapour diffusion of heptane into toluene. There are two crystallographically independent mol­ecules in the asymmetric unit, which are referred to as **14-A** [Fig. 17 ▸(*a*)] and **14-B** [Fig. 17 ▸(*b*)]. F_5_PhS-BsubPc arranges in the triclinic *P*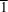 space group, with four mol­ecules in the unit cell [Fig. 17 ▸(*c*)]. Within the unit cell, there is concave–convex head-to-tail packing between **14-A** and **14-B**, with an inter­molecular distance of 3.958 Å. Upon extension of the unit cell, significant concave-to-axial ligand π–π inter­actions are observed between the π_Ph_ group and the π_ax_ group, with an inter­molecular distance of 3.525 Å. There is also an inter­action between the axial S atom and the π_ax_ group of an adjacent mol­ecule (π_ax_⋯S—B), at a distance of 3.560 Å. Finally, weak hy­dro­gen bonding is observed between an axial F atom and an adjacent π_Ph_ H atom (C—F_ax_⋯π_Ph_), at a distance of 2.546 Å. The inter­molecular inter­actions of F_5_PhS-BsubPc (**14**) are shown in Fig. 17 ▸(*d*).

**Figure 18:**
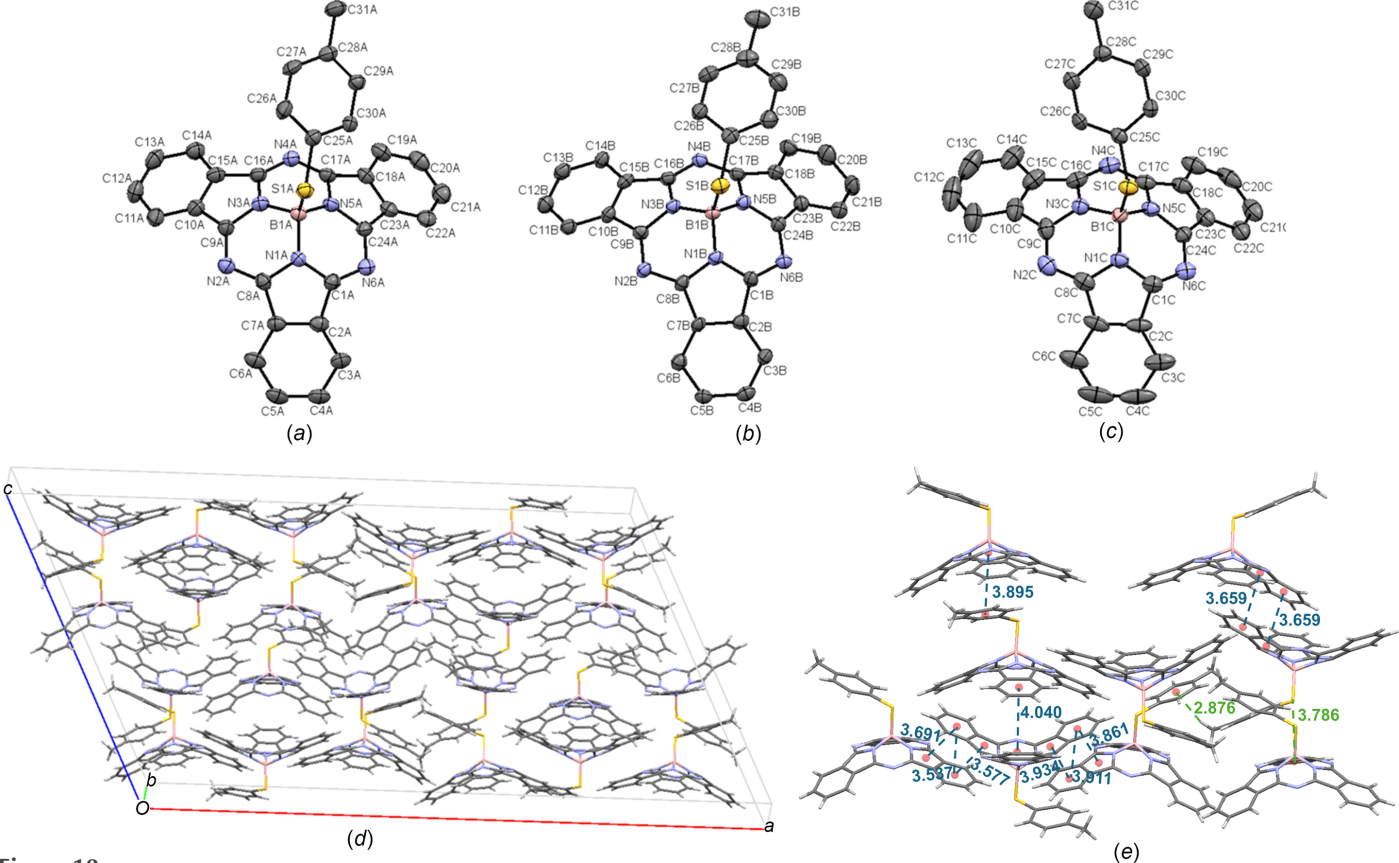
The mol­ecular structures of (*a*) MePhS-BsubPc mol­ecule *A* (**15-A**), (*b*) MePhS-BsubPc mol­ecule *B* (**15-B**) and (*c*) MePhS-BsubPc mol­ecule C (**15-C**), with displacement ellipsoids drawn at the 50% probability level and H atoms removed for clarity. (*d*) The unit-cell packing and (*e*) significant inter­molecular inter­actions in the crystal structure of **15**. π–π inter­actions are shown in blue and all other inter­molecular inter­actions are shown in green. Atom colours: carbon – gray, hy­dro­gen – white, nitro­gen – blue, boron – light pink and sulfur – orange.

MePhS-BsubPc (**15**) crystals were grown by slow vapour diffusion of heptane into toluene. There are three independent mol­ecules in the asymmetric unit that are referred to as **15-A** [Fig. 18 ▸(*a*)], **15-B** [Fig. 18 ▸(*b*)] and **15-C** [Fig. 18 ▸(*c*)]. MePhS-BsubPc arranges in the monoclinic *C*2/*c* space group, with 24 mol­ecules in the unit cell [Fig. 18 ▸(*d*)]. This is a different polymorph than the previously reported crystal structure of MePhS-BsubPc (Morse & Bender, 2012*b* ▸). With 24 mol­ecules in the unit cell, many inter­actions are observed. First, there are concave–concave head-to-head inter­actions between two adjacent **15-A** mol­ecules, with a π_pyr_–π_Ph_ distance of 3.659 Å. Next, there are less significant concave–concave head-to-tail inter­actions between two adjacent **15-B** mol­ecules at an inter­molecular distance of 4.040 Å. There is also a slightly offset convex–convex head-to-head inter­action between mol­ecules **15-B** and **15-C**, with inconsistent π_pyr_–π_Ph_ distances of 3.577 and 3.691 Å. The most significant overlap between iso­indole groups in this inter­action is between the adjacent π_Ph_ groups at a distance of 3.537 Å. A similar offset convex–convex head-to-head inter­action is seen between **15-A** and **15-B**, with π_pyr_–π_Ph_ distances of 3.861 and 3.934 Å, and a π_Ph_–π_Ph_ distance of 3.911 Å. There are also some significant inter­actions with the axial group. First, there is a concave-to-axial ligand inter­action between **15-B** and **15-C**, with a π–π inter­action observed between the axial arene group of **15-B** and the ‘tail’ of **15-C** at a distance of 3.895 Å. There is also a convex-to-axial ligand inter­action between the axial S atom of **15-A** and the π_Ph_ group of an adjacent **15-C** mol­ecule (π_Ph_⋯S—B) at a distance of 3.786 Å. Finally, there is a C_31_—H_ax_⋯π_ax_ inter­action between two adjacent **15-A** mol­ecules involving an axial methyl H atom and an axial arene group at a distance of 2.876 Å. The inter­molecular inter­actions of MePhS-BsubPc (**15**) are shown in Fig. 18 ▸(*e*).

**Figure 19:**
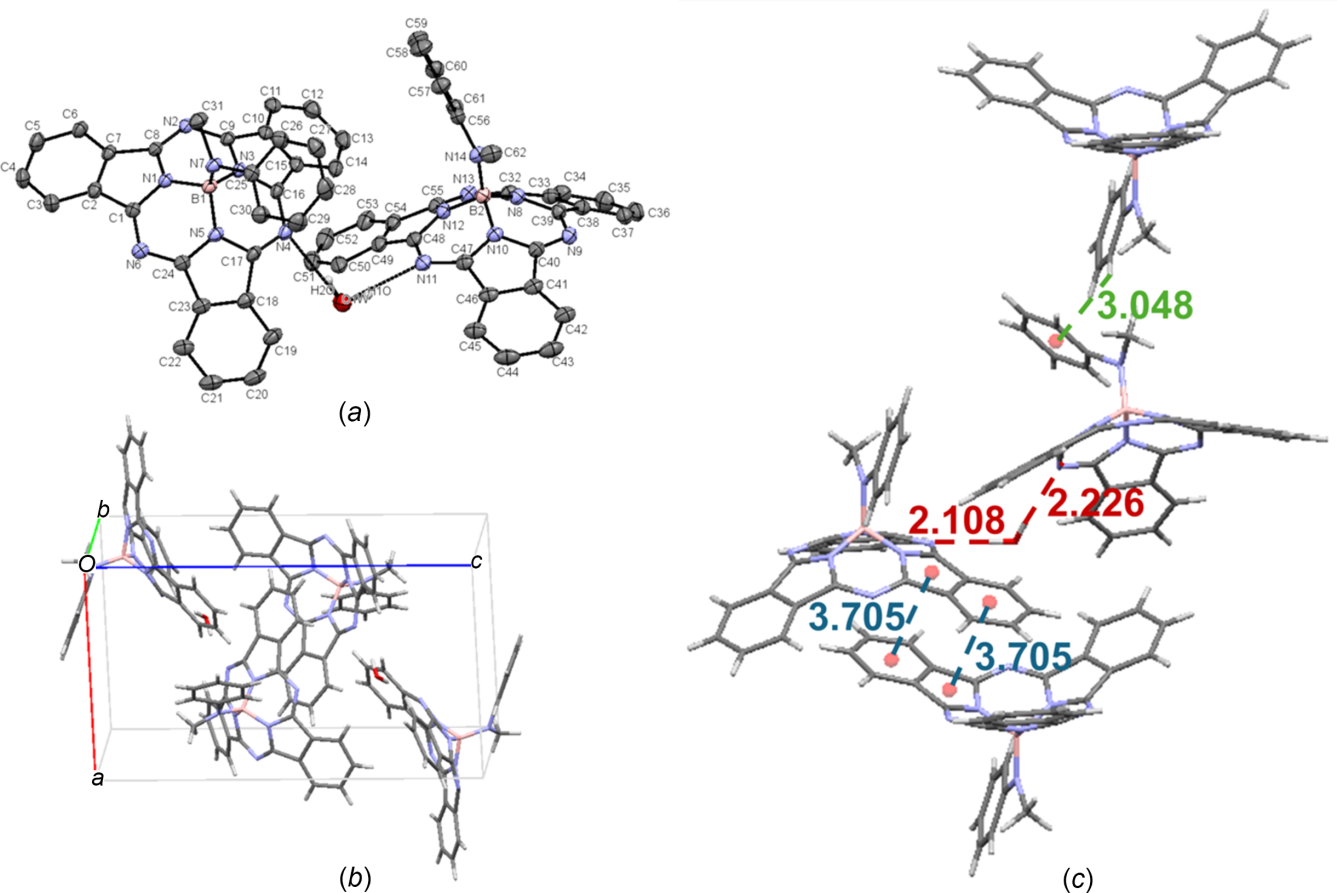
(*a*) The mol­ecular structure of PhMeN-BsubPc (**16-I**), with displacement ellipsoids drawn at the 50% probability level and H atoms removed for clarity, (*b*) the unit-cell packing and (*c*) significant inter­molecular inter­actions in the crystal structure. π–π inter­actions are shown in blue, hy­dro­gen bonding is shown in red and all other inter­molecular inter­actions are shown in green. Atom colours: carbon – gray, hy­dro­gen – white, nitro­gen – blue and boron – light pink.

**Figure 20:**
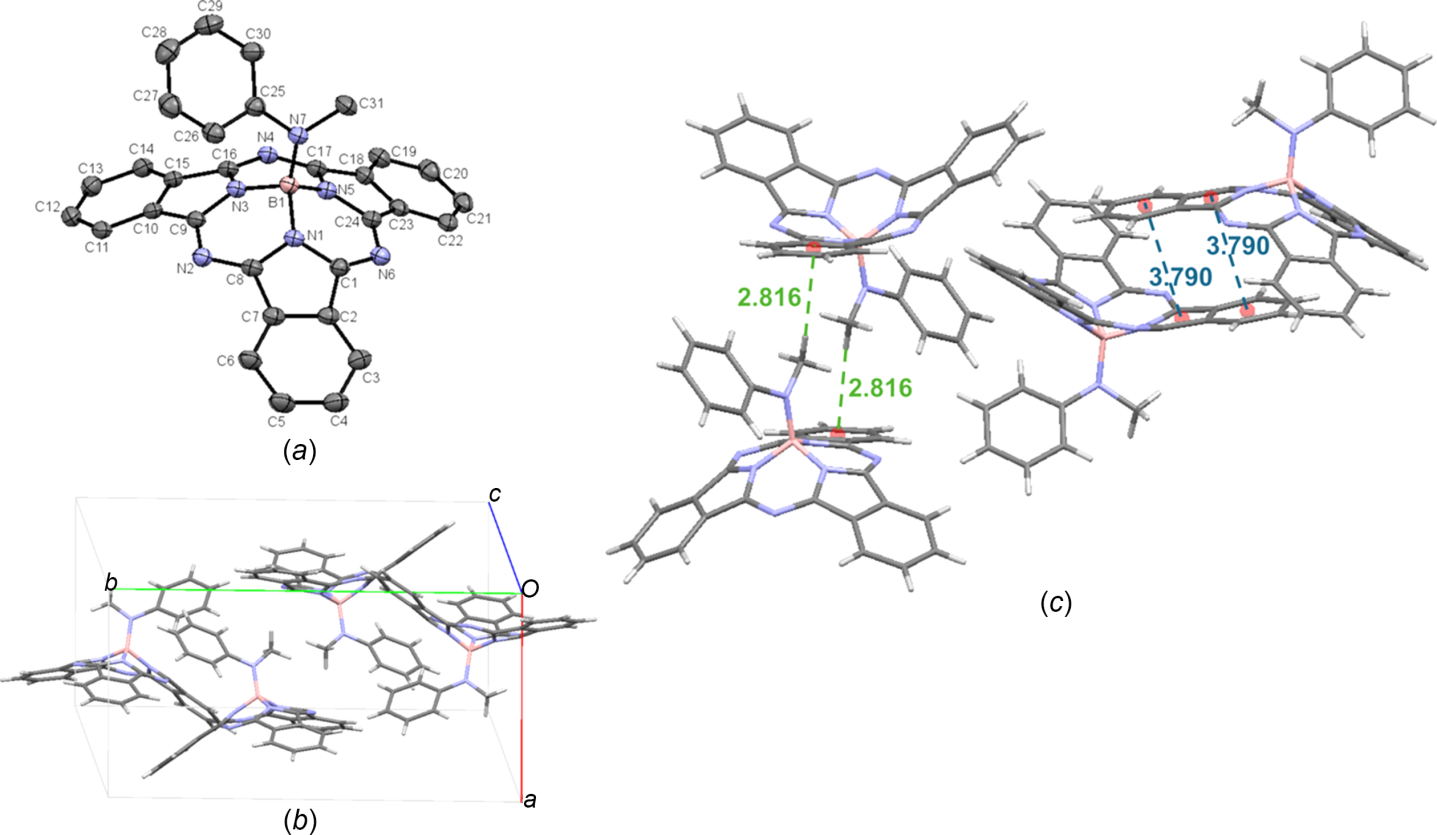
(*a*) The mol­ecular structure of PhMeN-BsubPc (**16-II**), with displacement ellipsoids drawn at the 50% probability level and H atoms removed for clarity, (*b*) the unit-cell packing and (*c*) significant inter­molecular inter­actions in the crystal structure. π–π inter­actions are shown in blue and all other inter­molecular inter­actions are shown in green. Atom colours: carbon – gray, hy­dro­gen – white, nitro­gen – blue and boron – light pink.

On two separate occasions, crystals of PhMeN-BsubPc were grown during a two-solvent recrystallization with THF and pentane, resulting in two different polymorphs. The first polymorph [**16-I**; Fig. 19 ▸(*a*)] crystallized in the triclinic *P*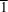 space group, with two mol­ecules in the asymmetric unit [**16-I** (mol 1) and **16-I** (mol 2)] and a partial occupancy water solvent mol­ecule [Fig. 19 ▸(*b*)], which is aligned with a previous report (Morse & Bender, 2012*b* ▸). The second polymorph [**16-II**; Fig. 20 ▸(*a*)] crystallized in the monoclinic *P*2_1_/*c* space group, with only one mol­ecule in the asymmetric unit and no water [Fig. 20 ▸(*b*)]. Both **16-I** and **16-II** have four mol­ecules in the unit cell. Concave–concave head-to-head inter­actions were seen within the unit cell for **16-I**, with a π_pyr_–π_Ph_ distance of 3.705 Å. There is also hy­dro­gen bonding between the water mol­ecule and the imine N atoms of two adjacent BsubPc mol­ecules. Upon extension of the unit cell, there is a C—H_Ph,ax_⋯π_ax_ inter­action between adjacent axial groups, with an inter­molecular distance of 3.048 Å. The inter­molecular inter­actions of **16-I** are shown in Fig. 19 ▸(*c*).

For another crystal of PhMeN-BsubPc (**16-II**), there are no significant π–π inter­actions between adjacent iso­indole units within the unit cell. The axial group appears to have a strong influence on the packing within the unit cell, with the most significant inter­actions being a convex-to-axial ligand C_31_—H_ax_⋯π_Ph_ inter­action between an axial methyl H atom and the adjacent π_Ph_ group (2.816 Å). Upon extension of the unit cell, there are concave–concave head-to-head inter­actions, with a π_Ph_–π_pyr_ distance of 3.790 Å, which is slightly longer than the analogous distance observed for **16-I**. The inter­molecular inter­actions of **16-II** are shown in Fig. 20 ▸(*c*).

#### Inter­molecular inter­actions summary

The π–π inter­actions observed in the crystal structures of the investigated BsubPcs are summarized in Table 2 ▸, and all inter­mole­cular inter­actions involving the axial group are sum­marized in Table 3 ▸. Concave–concave head-to-head inter­actions were the most common π–π inter­actions in the analyzed BsubPcs, observed in 10 of the 17 crystal structures (Table 2 ▸). Head-to-head inter­actions, whether concave–concave, convex–convex or concave–convex, tend to display closer packing than head-to-tail inter­actions. Only MeO- (**3**), naphth­oxy- (**10**), benzoate- (**12**) and F_5_PhS-BsubPc (**14**) didn’t display any head-to-head packing. Concave–convex inter­actions were rare, appearing only in the crystal structures of OctO- (**8**) and F_5_PhS-BsubPc (**14**).

Regarding inter­molecular inter­actions involving the axial group, convex-to-axial ligand inter­actions were quite common, appearing in nine of the investigated crystal structures. These inter­actions always involved a π_Ph_ group and either an axial H atom, which was common among the axial alk­oxy derivatives (**3**–**8**), or the axial heteroatom bonded to boron, in the cases of Cl- (**1**), Br- (**2**) and MePhS-BsubPc (**15**). F_3_EtO-BsubPc (**5**) was the only alk­oxy derivative that did not have any concave- or convex-to-axial ligand inter­actions. Its nonfluorinated analogue, EtO-BsubPc (**4**), displayed concave-to-axial ligand inter­actions between one of its axial methyl H atoms and an adjacent π_Ph_ group, but in the case of F_3_EtO-BsubPc (**5**), the axial methyl H atoms are all replaced by F atoms. The F atoms instead inter­acted with adjacent π_Ph_ H atoms through weak hy­dro­gen bonding. Furthermore, neither carboxyl­ate derivative (**11** and **12**) had any concave- or convex-to-axial ligand inter­actions. Both com­pounds have two O atoms in the axial group capable of weak hy­dro­gen bonding, which appeared to dictate the axial-group inter­actions.

Weak hy­dro­gen-bonding inter­actions were prevalent in com­pounds with boron–oxygen axial bonds (**3**–**13**). Of the alk­oxy derivatives (**3**–**8**), only tButO-BsubPc (**7**) did not display any hy­dro­gen bonding, likely due to steric effects. PhO-BsubPc (**9**) was the only other boron–oxygen-bonded com­pound that did not display any hy­dro­gen bonding. It looked like there may have been weak hy­dro­gen bonding between the axial O atom and an adjacent axial π_Ph_ H atom in the crystal structure of **9**, but the inter­molecular O⋯H distance (2.766 Å) was slightly greater than the sum of the van der Waals radii of oxygen and hy­dro­gen (2.72 Å). For the carboxyl­ate derivatives, acetate-BsubPc (**11**) had weak hy­dro­gen bonding involving both the axial O atom bonded to boron and the carbonyl O atom, whereas hy­dro­gen bonding was only seen with the carbonyl O atom of benzoate-BsubPc (**12**). Hydrogen bonding involving the axial N atom of PhMeN-BsubPc (**16**) was not observed for either polymorph.

Among the six com­pounds containing an arene group within the axial moiety (**9**, **10**, **12**, **14**, **15** and **16**), three had π–π inter­actions involving the axial group. Most significantly, the only π–π inter­actions seen for naphth­oxy-BsubPc (**10**) were concave-to-axial ligand π_ax_–π_tail_ inter­actions. The two thio­phen­oxy derivatives were the only other com­pounds to display π–π inter­actions involving the π_ax_ group. Inter­estingly, PhO-BsubPc (**9**) was the only investigated com­pound that didn’t have any inter­actions involving the axial group. Finally, the two polymorphs of PhMeN-BsubPc (**16**) have different inter­molecular inter­actions involving the axial group. Both have C—H_ax_⋯π inter­actions, but for **16-I** this inter­action is between an axial phenyl H atom and an adjacent π_ax_ group, while for **16-II** this inter­action is between an axial methyl H atom and an adjacent π_Ph_ group.

### Axial bond length and BsubPc bowl depth

**Figure 21:**
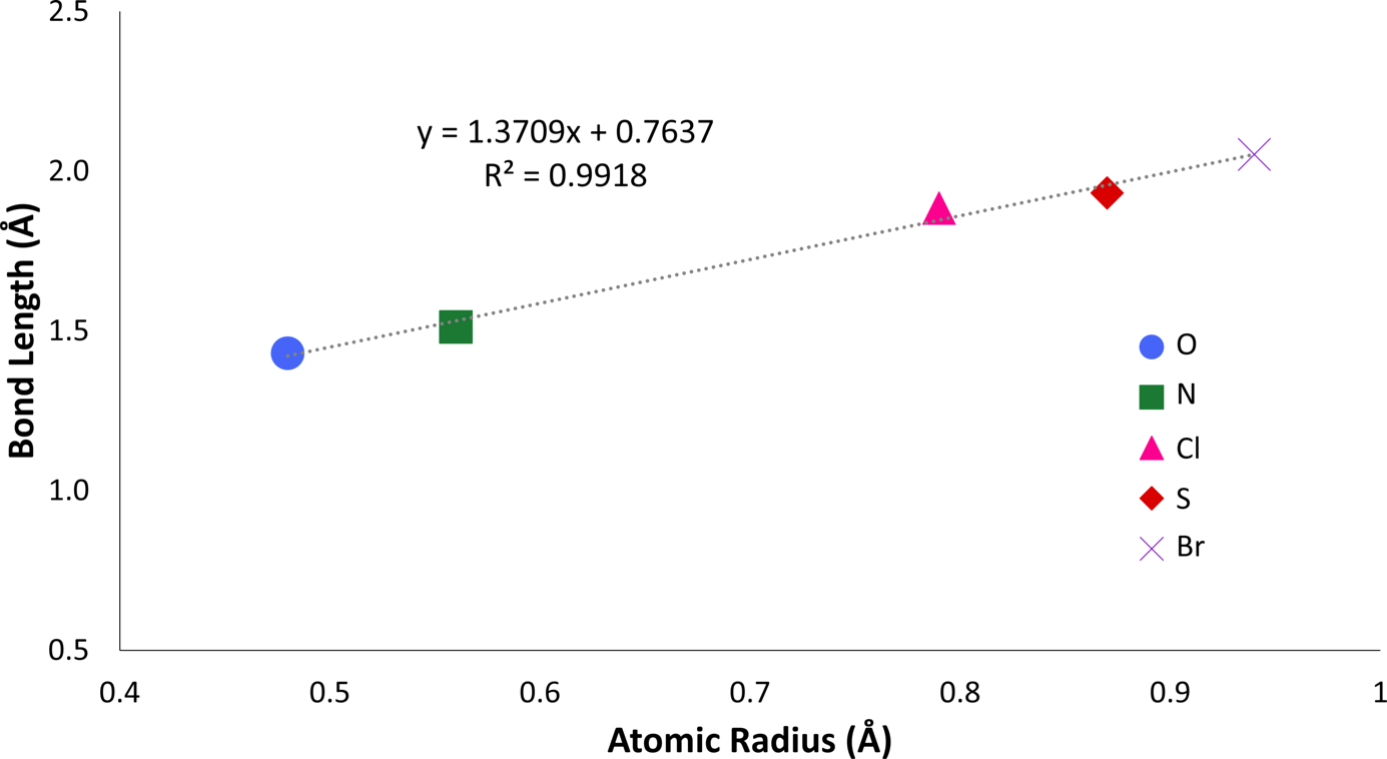
Atomic radius of the heteroatom bonded to boron (oxygen – blue circle, nitro­gen – green square, chlorine – pink triangle, sulfur – red diamond and bromine – purple X) *versus* the axial bond length. The boron–oxygen bond length is the average axial bond length of com­pounds **3**–**13** and the boron–sulfur bond length is the average axial bond length of com­pounds **14** and **15**.

The influence of the axial group on the axial bond length was also of inter­est in this study. The axial bond length is the distance between boron and the heteroatom to which it is bonded. As expected, the axial bond length increased linearly as the atomic radius of the heteroatom bonded to boron increased (Fig. 21 ▸). In cases where multiple com­pounds had the same heteroatom, the average bond length was used to generate Fig. 21 ▸. The average axial bond length was 1.430 ± 0.020 Å for boron–oxygen bonds, 1.512 ± 0.002 Å for boron–nitro­gen bonds, 1.884 Å for the boron–chlorine bond, 1.931 ± 0.021 Å for boron–sulfur bonds and 2.052 Å for the boron–bromine bond.

**Figure 22:**
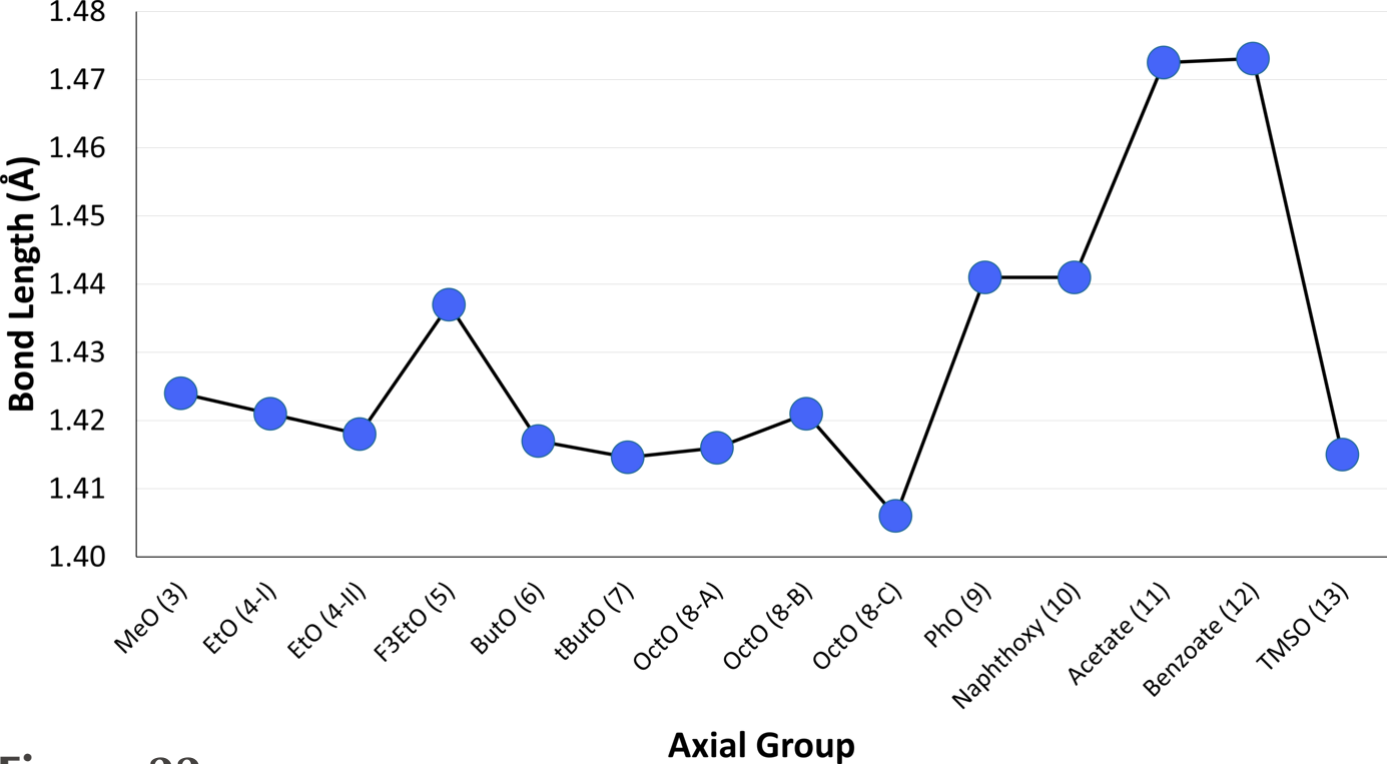
Axial bond lengths of the investigated BsubPcs with boron–oxygen axial bonds.

Additional trends were observed when com­paring the axial bond lengths of com­pounds with the same heteroatom. Within the com­pounds with boron–oxygen bonds (Fig. 22 ▸), the two carboxyl­ate derivatives [acetate- (**11**) and benzoate-BsubPc (**12**)] had the longest axial bond lengths at 1.473 Å for both com­pounds. The two phen­oxy derivatives [PhO- (**9**) and naphth­oxy-BsubPc (**10**)] had the next highest axial bond lengths at 1.441 Å for both com­pounds. For the axial alk­oxy derivatives (**3**–**8**), there was a general slight decrease in axial bond length as the length of the carbon chain increases. The exception was F_3_EtO-BsubPc (**5**), which had the longest axial bond length of the alk­oxy derivatives at 1.437 Å. The axial bond lengths of the three OctO-BsubPc (**8**) mol­ecules in the asymmetric unit showed some variation, resulting in an average bond length of 1.414 ± 0.006 Å. The bond length of TMSO-BsubPc (**13**) was most closely aligned with ButO-BsubPc (**6**), with both com­pounds having an axial bond length of 1.415 Å.

**Figure 23:**
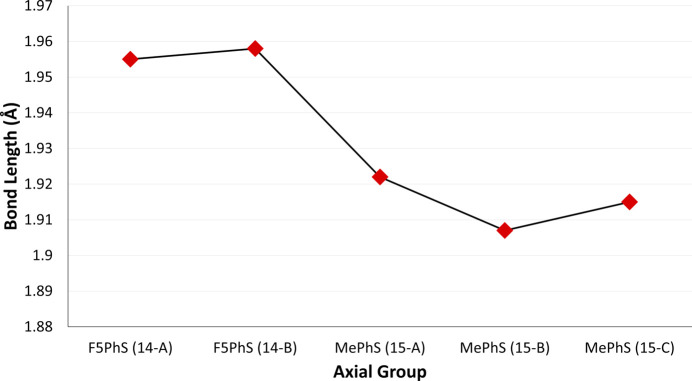
Axial bond lengths of the investigated BsubPcs with boron–sulfur axial bonds.

For the two com­pounds with axial boron–sulfur bonds, the axial bond length of F_5_PhS-BsubPc (**14**) was longer than that of MePhS-BsubPc (**15**) (Fig. 23 ▸). This is similar to what was seen with the alk­oxy derivatives (**3**–**8**), with the fluorine-containing axial group (**5**) having the longest axial bond length. These results indicate that F atoms in the axial group cause a slight lengthening of the axial bond. F_5_PhS-BsubPc (**14**) has two mol­ecules in its asymmetric unit, with an average bond length of 1.957 ± 0.001 Å, and MePhS-BsubPc (**15**) has three mol­ecules in its asymmetric unit, with an average bond length of 1.915 ± 0.006 Å.

**Figure 24:**
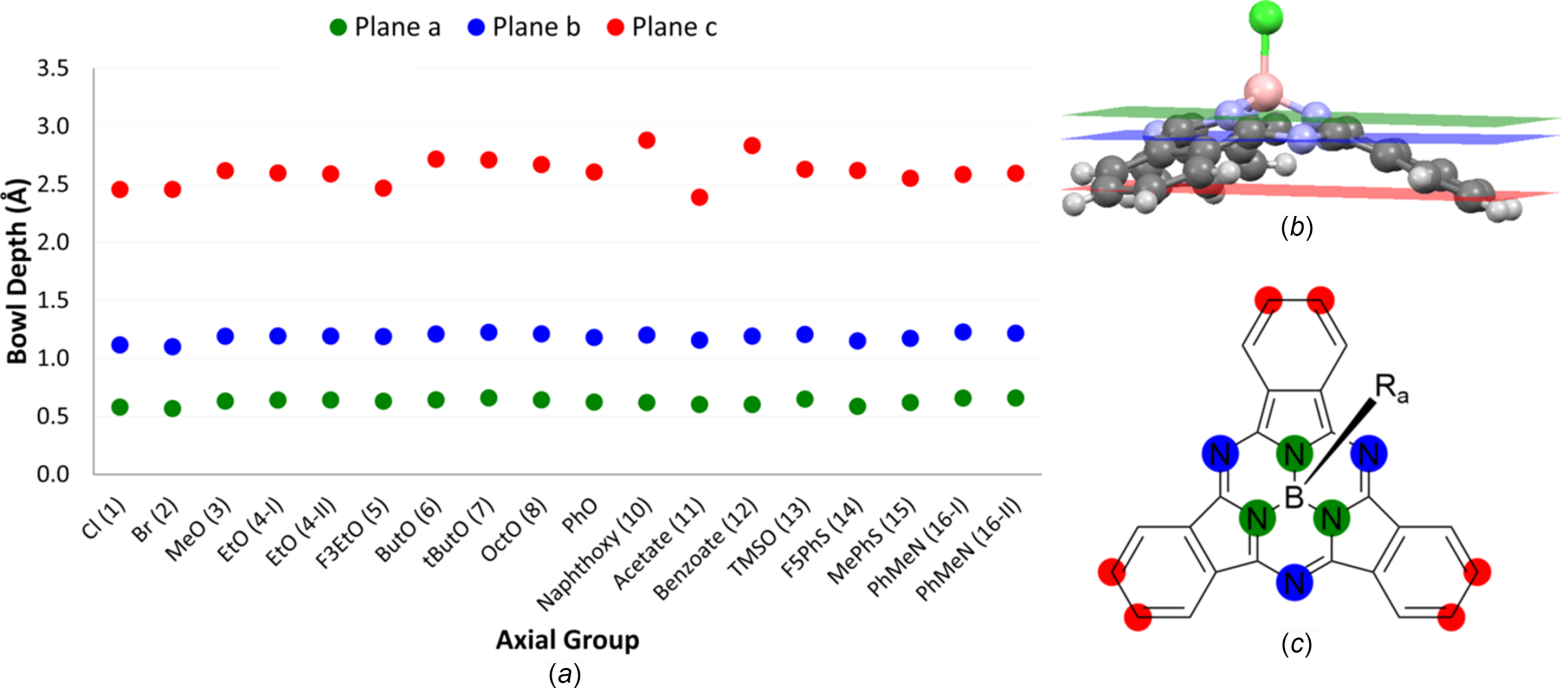
(*a*) Bowl depth variation with different axial substituents. Three bowl depths are reported as the shortest distance between boron and plane *a* (green), boron and plane *b* (blue), and boron and plane *c* (red). (*b*) 3D structure of Cl-BsubPc defining planes *a*, *b* and *c*, and (*c*) 2D general BsubPc structure highlighting the atoms that define planes *a*, *b* and *c*. For com­pounds with multiple mol­ecules in the asymmetric unit (**8**, **14**, **15** and **16-I**), the reported bowl depths are an average of all the mol­ecules in the asymmetric unit.

Bowl depth is a measure of the degree of nonplanarity of the BsubPc macrocycle. The greater the bowl depth, the less planar the mol­ecule. Three bowl depths were calculated for each BsubPc (Fig. 24 ▸) by measuring the distance between the B atom and (i) the plane defined by the three pyrrole N atoms (plane *a*, green), (ii) the plane defined by the three imine N atoms (plane *b*, blue), and (iii) the plane defined by the six outermost C atoms (plane *c*, red). The greatest variation in bowl depth across the axially substituted BsubPcs was seen when considering the distance between boron and plane *c* (Fig. 24 ▸). Very little variation was seen in bowl depth when considering the distance between boron and planes *a* and *b* (Fig. 24 ▸). Therefore, this discussion will focus on the bowl depth that is measured between boron and plane *c*.

Unlike for the axial bond length, the nature of the heteroatom bonded to boron did not have a clear influence on the BsubPc bowl depth. The axially halogenated derivatives [Cl- (**1**) and Br-BsubPc (**2**)] had two of the smallest bowl depths of the investigated com­pounds, although the nature of the halogen atom had an almost negligible impact. Acetate-BsubPc (**11**) was the only com­pound with a smaller bowl depth than the halogens and had the smallest bowl depth of all investigated com­pounds. Inter­estingly, the other investigated carboxyl­ate derivative, benzoate-BsubPc (**12**), had the second largest bowl depth of all investigated BsubPcs, behind only naphth­oxy-BsubPc (**10**). It appears that adding arenes to otherwise identical axial substituents increases the bowl depth. For instance, naphth­oxy-BsubPc (**10**), which has two arene sites in the axial position, has a larger bowl depth than PhO-BsubPc (**9**), which has one arene site in the axial position. Furthermore, benzoate-BsubPc (**12**) has a larger bowl depth than acetate-BsubPc (**11**), with the only difference being an arene in place of a methyl group.

While F_3_EtO-BsubPc (**5**) stuck out from the other alk­oxy-BsubPc derivatives (**3**–**8**) with the longest bond length, it stuck out again here with the smallest bowl depth. Among the nonfluorinated alk­oxy derivatives, there does not appear to be a clear correlation between the bowl depth and the length of the axial carbon chain. Furthermore, there was a minimal difference in the bowl depths of ButO- (**6**) and tButO-BsubPc (**7**), indicating that branching does not impact the bowl depth. However, the bowl depth of TMSO-BsubPc (**13**) was smaller than that of its structural analogue, tButO-BsubPc (**7**), indicating that the presence of an Si atom in place of a C atom has a modest impact on the planarity of the BsubPc.

The thio­phen­oxy derivatives (**14** and **15**) both have multiple mol­ecules in their asymmetric units with varying bowl depths. For F_5_PhS-BsubPc (**14**), there are two independent mol­ecules in the asymmetric unit, with an average bowl depth of 2.620 ± 0.131 Å. For MePhS-BsubPc (**15**), the average bowl depth of the three mol­ecules is 2.553 ± 0.019 Å. Therefore, adding F atoms to the thio­phen­oxy axial group increases both the BsubPc bowl depth and the axial bond length, in contrast to what was seen when adding F atoms to the axial alk­oxy group, whereby the bond length increased and the bowl depth decreased.

Finally, the two PhMeN-BsubPc (**16**) polymorphs have very similar bowl depths when com­paring the average bowl depth of the two mol­ecules in **16-I** (2.586 ± 0.45 Å) to the bowl depth of **16-II** (2.595 Å).

The axial bond lengths and BsubPc bowl depths are tabulated in Table 4 ▸.

## Conclusions

Overall, the axial-group inter­actions play a significant role in the crystal packing of BsubPcs, influencing the crystal system/space group and inter­molecular inter­actions. Concave–concave head-to-head inter­actions were the most common π–π inter­actions in the crystal structures of the investigated BsubPcs, whereas axial-group inter­actions with adjacent isoindoline units tended to favour the convex side of the bowl. Weak hy­dro­gen-bonding inter­actions are prevalent when the axial group contains O and/or F atoms, but the axial N atom in PhMeN-BsubPc did not participate in hy­dro­gen bonding. This may be a result of steric hindrance, as other investigated com­pounds with bulky axial groups, such as tButO-BsubPc and OctO-BsubPc, also disrupted some of the inter­molecular inter­actions. The bulkiness of tButO-BsubPc disrupted hy­dro­gen bonding with the axial O atom, while OctO-BsubPc disrupted the inter­molecular π–π inter­actions. The presence of arenes in the axial moiety can also have a significant impact, as π–π inter­actions involving the axial group are now possible. In particular, naphth­oxy-, F_5_PhS- and MePhS-BsubPc all have concave-to-axial π–π inter­actions. Furthermore, the axial bond length increases linearly with the atomic radius of the heteroatom bonded to boron, and the presence of F atoms in the axial group tended to lengthen the axial bond. However, there is no clear trend in BsubPc bowl depth related to the axial group, with only a modest impact being observed. There were also no clear trends observed based on some of the crystal growth methods. The crystal structures of EtO-BsubPc from train sublimation (**4-I**) and slow evaporation of acetone (**4-II**) were identical, as were the crystal structures of acetate- (**11**) and benzoate-BsubPc (**12**) grown from train sublimation in this study com­pared to a previous report of the crystal structures from solvent-based crystal growth methods. Train sublimation is often the preferred crystal growth method, as it most closely mimics the vacuum deposition conditions used for the thin film fabrication in organic electronic applicants. However, in this case, the solvent-based crystal growth methods appeared to produce the same results as train sublimation. The only observed solvent effects were when hygroscopic solvents methanol and THF were used to grow crystals of OctO- (**8**) and PhMeN-BsubPc (**16-I**), respectively, with water mol­ecules incorporated into the crystal structures and hy­dro­gen bonding with imine N atoms. Overall, the results of this study are useful for the nanoengineering of thin films of BsubPcs for their application in organic electronics.

## Supplementary Material

Crystal structure: contains datablock(s) d2344a_a, d23119a_a, d2368_a, d22127_a, d22128_a, d22126_a, d2365_a, d23115_a, d23123_a, d2335_a, d23108a_a, d22121a_a, d22123a_a, d23117_a, d23110_a, d23112_a_tw_sq, d2339_a, d22150_a, global. DOI: 10.1107/S2053229624006934/dg3059sup1.cif

Structure factors: contains datablock(s) d2344a_a. DOI: 10.1107/S2053229624006934/dg3059d2344a_asup2.hkl

Structure factors: contains datablock(s) d23119a_a. DOI: 10.1107/S2053229624006934/dg3059d23119a_asup3.hkl

Structure factors: contains datablock(s) d2368_a. DOI: 10.1107/S2053229624006934/dg3059d2368_asup4.hkl

Structure factors: contains datablock(s) d22127_a. DOI: 10.1107/S2053229624006934/dg3059d22127_asup5.hkl

Structure factors: contains datablock(s) d22128_a. DOI: 10.1107/S2053229624006934/dg3059d22128_asup6.hkl

Structure factors: contains datablock(s) d22126_a. DOI: 10.1107/S2053229624006934/dg3059d22126_asup7.hkl

Structure factors: contains datablock(s) d2365_a. DOI: 10.1107/S2053229624006934/dg3059d2365_asup8.hkl

Structure factors: contains datablock(s) d23115_a. DOI: 10.1107/S2053229624006934/dg3059d23115_asup9.hkl

Structure factors: contains datablock(s) d23123_a. DOI: 10.1107/S2053229624006934/dg3059d23123_asup10.hkl

Structure factors: contains datablock(s) d2335_a. DOI: 10.1107/S2053229624006934/dg3059d2335_asup11.hkl

Structure factors: contains datablock(s) d23108a_a. DOI: 10.1107/S2053229624006934/dg3059d23108a_asup12.hkl

Structure factors: contains datablock(s) d22121a_a. DOI: 10.1107/S2053229624006934/dg3059d22121a_asup13.hkl

Structure factors: contains datablock(s) d22123a_a. DOI: 10.1107/S2053229624006934/dg3059d22123a_asup14.hkl

Structure factors: contains datablock(s) d23117_a. DOI: 10.1107/S2053229624006934/dg3059d23117_asup15.hkl

Structure factors: contains datablock(s) d23110_a. DOI: 10.1107/S2053229624006934/dg3059d23110_asup16.hkl

Structure factors: contains datablock(s) d23112_a_tw_sq. DOI: 10.1107/S2053229624006934/dg3059d23112_a_tw_sqsup17.hkl

Structure factors: contains datablock(s) d2339_a. DOI: 10.1107/S2053229624006934/dg3059d2339_asup18.hkl

Structure factors: contains datablock(s) d22150_a. DOI: 10.1107/S2053229624006934/dg3059d22150_asup19.hkl

CCDC references: 2363939, 2363938, 2363937, 2363936, 2363935, 2363934, 2363933, 2363932, 2363931, 2363930, 2363929, 2363928, 2363927, 2363926, 2363925, 2363924, 2363923, 2363922

## Figures and Tables

**Table:** Experiments were carried out using a Bruker Kappa APEX DUO CMOS Photon II diffractometer at 150 K. H-atom parameters were constrained. Absorption was corrected for by multi-scan methods (*SADABS*; Krause *et al.*, 2015 ▸).

	Cl-BsubPc (**1**)	Br-BsubPc (**2**)	MeO-BsubPc (**3**)	EtO-BsubPc (**4-I**)
CCDC Deposition No.	2363922	2363923	2363924	2363925
Crystallization method	Sublimation (405 °C)	Sublimation (350 °C)	Sublimation (300 °C)	Sublimation (295 °C)
Crystal data				
Chemical formula	C_24_H_12_BClN_6_	C_24_H_12_BBrN_6_	C_25_H_15_BN_6_O	C_26_H_17_BN_6_O
*M*_r_	430.66	475.12	426.24	440.26
Crystal system, space group	Orthorhombic, *Pnma*	Orthorhombic, *Pnma*	Orthorhombic, *Pnma*	Monoclinic, *P*2_1_/*n*
*a*, *b*, *c* (Å)	12.0315 (10), 14.8174 (13), 10.3002 (8)	12.0010 (6), 15.0617 (8), 10.3879 (5)	12.3162 (11), 15.2325 (19), 10.4151 (10)	9.0313 (14), 14.220 (2), 16.474 (2)
α, β, γ (°)	90, 90, 90	90, 90, 90	90, 90, 90	90, 102.667 (4), 90
*V* (Å^3^)	1836.3 (3)	1877.67 (16)	1953.9 (4)	2064.3 (5)
*Z*	4	4	4	4
Radiation type	Cu *K*α	Mo *K*α	Cu *K*α	Mo *K*α
μ (mm^−1^)	2.07	2.22	0.75	0.09
Crystal size (mm)	0.15 × 0.06 × 0.02	0.27 × 0.16 × 0.14	0.10 × 0.04 × 0.02	0.21 × 0.12 × 0.03

Data collection				
*T*_min_, *T*_max_	0.646, 0.753	0.688, 0.746	0.659, 0.753	0.684, 0.746
No. of measured, independent and observed [*I* > 2σ(*I*)] reflections	19930, 1647, 1416	23127, 2243, 1955	27808, 1762, 1570	49575, 4744, 3311
*R*_int_	0.061	0.027	0.057	0.101
(sin θ/λ)_max_ (Å^−1^)	0.593	0.650	0.592	0.650

Refinement				
*R*[*F*^2^ > 2σ(*F*^2^)], *wR*(*F*^2^), *S*	0.035, 0.097, 1.06	0.021, 0.057, 1.09	0.032, 0.078, 1.08	0.047, 0.098, 1.04
No. of reflections	1647	2243	1762	4744
No. of parameters	151	151	159	308
No. of restraints	0	0	0	0
Δρ_max_, Δρ_min_ (e Å^−3^)	0.24, −0.35	0.38, −0.26	0.22, −0.19	0.21, −0.23

**Table:** 

	EtO-BsubPc (**4-II**)	F_3_EtO-BsubPc (**5**)	ButO-BsubPc (**6**)	tButO-BsubPc (**7**)
CCDC Deposition No.	2363926	2363927	2363928	2363929
Crystallization method	Slow evaporation – acetone	Sublimation (305 °C)	Sublimation (265 °C)	Rotovap – DCM/hexanes
Crystal data				
Chemical formula	C_26_H_17_BN_6_O	C_26_H_14_BF_3_N_6_O	C_28_H_21_BN_6_O	C_28_H_21_BN_6_O
*M*_r_	440.26	494.24	468.32	468.32
Crystal system, space group	Monoclinic, *P*2_1_/*n*	Monoclinic, *P*2_1_/*c*	Orthorhombic, *Pbca*	Monoclinic, *P*2_1_/*c*
*a*, *b*, *c* (Å)	9.0304 (5), 14.2423 (8), 16.4452 (9)	10.4664 (6), 15.4986 (8), 14.3171 (8)	14.6398 (8), 15.2842 (9), 20.5313 (12)	14.3815 (7), 8.2702 (4), 19.8660 (9)
α, β, γ (°)	90, 102.476 (3), 90	90, 110.221 (3), 90	90, 90, 90	90, 102.074 (2), 90
*V* (Å^3^)	2065.1 (2)	2179.3 (2)	4594.0 (5)	2310.55 (19)
*Z*	4	4	8	4
Radiation type	Cu *K*α	Cu *K*α	Cu *K*α	Cu *K*α
μ (mm^−1^)	0.72	0.96	0.68	0.68
Crystal size (mm)	0.15 × 0.15 × 0.10	0.14 × 0.09 × 0.04	0.15 × 0.07 × 0.06	0.17 × 0.09 × 0.02

Data collection				
*T*_min_, *T*_max_	0.654, 0.753	0.624, 0.753	0.661, 0.753	0.673, 0.753
No. of measured, independent and observed [*I* > 2σ(*I*)] reflections	46104, 3555, 3148	39716, 3759, 3043	45733, 3951, 3295	41764, 3994, 3363
*R*_int_	0.059	0.083	0.065	0.052
(sin θ/λ)_max_ (Å^−1^)	0.593	0.594	0.594	0.594

Refinement				
*R*[*F*^2^ > 2σ(*F*^2^)], *wR*(*F*^2^), *S*	0.039, 0.102, 1.05	0.039, 0.107, 1.04	0.038, 0.095, 1.07	0.035, 0.089, 1.05
No. of reflections	3555	3759	3951	3994
No. of parameters	308	335	326	329
No. of restraints	0	0	0	0
Δρ_max_, Δρ_min_ (e Å^−3^)	0.50, −0.21	0.27, −0.28	0.33, −0.22	0.22, −0.17

**Table:** 

	OctO-BsubPc (**8**)	PhO-BsubPc (**9**)	Naphthoxy-BsubPc (**10**)	Acetate-BsubPc (**11**)
CCDC Deposition No.	2363930	2363931	2363932	2363933
Crystallization method	Slow Evaporation – methanol	SVD – toluene/heptane	Sublimation (315 °C)	Sublimation (290 °C)
Crystal data				
Chemical formula	6C_32_H_29_BN_6_O·0.5H_2_O	C_30_H_17_BN_6_O	C_34_H_19_BN_6_O	C_26_H_15_BN_6_O_2_
*M*_r_	3166.55	488.30	538.36	454.25
Crystal system, space group	Triclinic, *P* 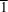	Triclinic, *P* 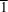	Orthorhombic, *Pnma*	Triclinic, *P* 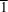
*a*, *b*, *c* (Å)	15.3028 (7), 16.2278 (7), 17.3604 (7)	10.0268 (10), 10.7263 (12), 11.8090 (13)	17.133 (3), 13.929 (2), 10.3669 (16)	9.3432 (9), 9.3548 (7), 12.3322 (12)
α, β, γ (°)	103.045 (3), 105.716 (3), 94.022 (3)	85.879 (4), 77.440 (3), 66.151 (3)	90, 90, 90	104.203 (2), 100.751 (3), 90.701 (2)
*V* (Å^3^)	4003.0 (3)	1133.6 (2)	2474.0 (7)	1024.69 (16)
*Z*	1	2	4	2
Radiation type	Cu *K*α	Mo *K*α	Mo *K*α	Mo *K*α
μ (mm^−1^)	0.65	0.09	0.09	0.10
Crystal size (mm)	0.24 × 0.16 × 0.06	0.28 × 0.15 × 0.09	0.26 × 0.15 × 0.08	0.24 × 0.22 × 0.13

Data collection				
*T*_min_, *T*_max_	0.622, 0.753	0.687, 0.746	0.609, 0.746	0.710, 0.746
No. of measured, independent and observed [*I* > 2σ(*I*)] reflections	110436, 13816, 10618	26461, 5137, 3795	17347, 2963, 1878	21123, 4670, 3665
*R*_int_	0.070	0.052	0.092	0.038
(sin θ/λ)_max_ (Å^−1^)	0.594	0.649	0.650	0.650

Refinement				
*R*[*F*^2^ > 2σ(*F*^2^)], *wR*(*F*^2^), *S*	0.054, 0.149, 1.05	0.039, 0.095, 1.03	0.052, 0.119, 1.05	0.038, 0.096, 1.03
No. of reflections	13816	5137	2963	4670
No. of parameters	1201	343	217	317
No. of restraints	499	0	0	0
Δρ_max_, Δρ_min_ (e Å^−3^)	0.61, −0.39	0.22, −0.23	0.26, −0.28	0.32, −0.28

**Table:** 

	Benzoate-BsubPc (**12**)	TMSO-BsubPc (**13**)	F_5_PhS-BsubPc (**14**)	MePhS-BsubPC (**15**)
CCDC Deposition No.	2363934	2363935	2363936	2363937
Crystallization method	Sublimation (355 °C)	Rotovap – DCM/hexanes	SVD – toluene/heptane	SVD – toluene/heptane
Crystal data				
Chemical formula	C_31_H_17_BN_6_O_2_	C_27_H_21_BN_6_OSi	C_30_H_12_BF_5_N_6_S	C_31_H_19_BN_6_S
*M*_r_	516.31	484.40	594.33	518.39
Crystal system, space group	Monoclinic, *P*2_1_/*c*	Monoclinic, *P*2_1_/*c*	Triclinic, *P* 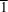	Monoclinic, *C*2/*c*
*a*, *b*, *c* (Å)	15.671 (2), 11.1920 (13), 15.2363 (19)	16.343 (3), 11.9655 (16), 12.5059 (19)	10.7010 (5), 11.8651 (6), 22.4968 (13)	49.653 (4), 12.1268 (10), 27.559 (2)
α, β, γ (°)	90, 115.386 (4), 90	90, 99.996 (11), 90	95.863 (2), 92.861 (2), 115.433 (2)	90, 114.838 (4), 90
*V* (Å^3^)	2414.3 (5)	2408.4 (6)	2552.1 (2)	15059 (2)
*Z*	4	4	4	24
Radiation type	Mo *K*α	Cu *K*α	Mo *K*α	Cu *K*α
μ (mm^−1^)	0.09	1.13	0.20	1.41
Crystal size (mm)	0.30 × 0.15 × 0.12	0.19 × 0.15 × 0.01	0.35 × 0.24 × 0.05	0.28 × 0.27 × 0.02

Data collection				
*T*_min_, *T*_max_	0.685, 0.746	0.629, 0.753	0.713, 0.746	0.633, 0.753
No. of measured, independent and observed [*I* > 2σ(*I*)] reflections	49333, 5530, 4050	57016, 4230, 2990	75917, 11711, 7793	12819, 12819, 10107
*R*_int_	0.071	0.127	0.073	0.091
(sin θ/λ)_max_ (Å^−1^)	0.650	0.608	0.650	0.594

Refinement				
*R*[*F*^2^ > 2σ(*F*^2^)], *wR*(*F*^2^), *S*	0.040, 0.095, 1.03	0.054, 0.123, 1.05	0.043, 0.102, 1.02	0.098, 0.242, 1.09
No. of reflections	5530	4230	11711	12819
No. of parameters	361	326	775	1059
No. of restraints	0	0	0	0
Δρ_max_, Δρ_min_ (e Å^−3^)	0.23, −0.24	0.31, −0.24	0.39, −0.31	0.89, −0.57

**Table:** 

	PhMeN-BsubPc (**16-I**)	PhMeN-BsubPc (**16-II**)
CCDC Deposition No.	2363938	2363939
Crystallization method	Recrystallization – THF/pentane	Recrystallization – THF/pentane
Crystal data		
Chemical formula	C_31_H_20_BN_7_·0.14H_2_O	C_31_H_20_BN_7_
*M*_r_	503.87	501.35
Crystal system, space group	Triclinic, *P* 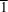	Monoclinic, *P*2_1_/*c*
*a*, *b*, *c* (Å)	11.4897 (5), 11.7489 (5), 19.3046 (8)	10.3502 (4), 20.7509 (8), 12.2816 (5)
α, β, γ (°)	73.348 (2), 81.941 (2), 76.167 (2)	90, 114.734 (2), 90
*V* (Å^3^)	2417.05 (18)	2395.80 (17)
*Z*	4	4
Radiation type	Cu *K*α	Cu *K*α
μ (mm^−1^)	0.68	0.68
Crystal size (mm)	0.20 × 0.15 × 0.08	0.19 × 0.14 × 0.09

Data collection		
*T*_min_, *T*_max_	0.657, 0.753	0.462, 0.753
No. of measured, independent and observed [*I* > 2σ(*I*)] reflections	60606, 8275, 7193	41606, 4159, 3432
*R*_int_	0.046	0.111
(sin θ/λ)_max_ (Å^−1^)	0.592	0.595

Refinement		
*R*[*F*^2^ > 2σ(*F*^2^)], *wR*(*F*^2^), *S*	0.037, 0.093, 1.06	0.055, 0.165, 1.06
No. of reflections	8275	4159
No. of parameters	713	354
No. of restraints	0	0
Δρ_max_, Δρ_min_ (e Å^−3^)	0.27, −0.29	0.28, −0.35

Computer programs: *APEX4* (Bruker, 2021 ▸), *SHELXT2018* (Sheldrick, 2015*a* ▸), *SHELXL2019* (Sheldrick, 2015*b* ▸), *PLATON* (Spek, 2020 ▸) and *SHELXTL* (Sheldrick, 2008 ▸).

**Table 2:** Summary of π–π inter­actions (Å), not including any axial-group inter­actions

Axial group	Concave–concave		Convex–convex			Concave–convex	
	Head-to-head	Head-to-tail	Head-to-head	Head-to-tail	Tail-to-tail	Head-to-head	Head-to-tail
Cl (**1**)	–	4.150	3.615*^*a*^*	–	–	–	–
Br (**2**)	–	4.151	3.661*^*a*^*	–	–	–	–
MeO (**3**)	–	4.044	–	–	–	–	–
EtO (**4-I**)	3.574*^*a*^*	–	–	–	–	–	–
EtO (**4-II**)	3.577*^*a*^*	–	–	–	–	–	–
F_3_EtO (**5**)	3.640*^*a*^*	–	–	3.822	–	–	–
ButO (**6**)	3.612*^*a*^*	–	–	4.295	–	–	–
tButO (**7**)	3.564*^*a*^*	–	–	–	–	–	–
OctO (**8**)	–	–	–	–	–	3.628*^*a*^*, 3.673*^*a*^*	3.890
PhO (**9**)	3.674*^*a*^*	–	–	–	–	–	–
Naphth­oxy (**10**)	–	–	–	–	–	–	–
Acetate (**11**)	3.620*^*a*^*	–	–	–	3.897	–	–
Benzoate (**12**)	–	–	–	4.163	–	–	–
TMSO (**13**)	3.543*^*a*^*	–	–	–	–	–	–
F_5_PhS (**14**)	–	–	–	–	–	–	3.958
MePhS (**15**)	3.659*^*a*^*	4.040	3.577*^*a*^*	–	–	–	–
			3.691*^*a*^*				
			3.537*^*b*^*				
			3.861*^*a*^*				
			3.934*^*a*^*				
			3.911*^*b*^*				
PhMeN (**16-I**)	3.705*^*a*^*	–	–	–	–	–	–
PhMeN (**16-II**)	3.790*^*a*^*	–	–	–	–	–	–

Notes: (*a*) π_pyr_–π_Ph_ distance; (*b*) π_Ph_–π_Ph_ distance.

**Table 3:** Summary of inter­molecular inter­actions (Å) involving the axial group

Axial group	Convex-to-axial ligand		Concave-to-axial ligand			Hydrogen/halogen bonding				π_ax_ inter­actions		
	B—*A*⋯π_Ph_	C—H_ax_⋯π_Ph_	π_ax_–π_tail_	π_ax_–π_Ph_	C—H_ax_⋯π_Ph_	B—*A*⋯H_Ph_—C	C—H_ax_⋯N_imine_	C—F_ax_⋯H_Ph_—C	C=O_ax_⋯H_Ph_—C	C—H_Ph_⋯π_ax_	B—*A*⋯π_ax_	C—H_ax_⋯π_ax_
Cl (**1**)	3.548	–	–	–	–	2.893*^*b*^*	–	–	–	–	–	–
Br (**2**)	3.475	–	–	–	–	2.977*^*b*^*	–	–	–	–	–	–
MeO (**3**)	–	2.605	–	–	–	2.637*^*a*^*	–	–	–	–	–	–
EtO (**4-I**)	–	–	–	–	3.593	2.682*^*a*^*	–	–	–	–	–	–
EtO (**4-II**)	–	–	–	–	3.590	2.690*^*a*^*	–	–	–	–	–	–
F_3_EtO (**5**)	–	–	–	–	–	2.446*^*a*^*	2.622*^*a*^*	2.529*^*a*^*	–	–	–	–
ButO (**6**)	–	3.319	–	–	–	2.556*^*a*^*	2.640*^*a*^*	–	–	–	–	–
tButO (**7**)	–	3.057	–	–	–	–	–	–	–	–	–	–
OctO (**8**)	–	2.971	–	–	–	2.641*^*a*^*	–	–	–	–	–	–
PhO (**9**)	–	–	–	–	–	–	–	–	–	–	–	–
Naphth­oxy (**10**)	–	–	3.813	–	–	2.627*^*a*^*	–	–	–	–	–	–
Acetate (**11**)	–	–	–	–	–	2.581*^*a*^*	2.635*^*a*^*	–	2.628*^*a*^*	–	–	–
Benzoate (**12**)	–	–	–	–	–	–	–	–	2.510*^*a*^*, 2.536*^*a*^*	2.726, 3.612	–	–
TMSO (**13**)	–	3.070	–	–	–	2.478*^*a*^*	–	–	–	–	–	–
F_5_PhS (**14**)	–	–	–	3.525	–	–	–	2.546*^*a*^*	–	–	3.560	–
MePhS (**15**)	3.786	–	3.895	–	–	–	–	–	–	–	–	2.876
PhMeN (**16-I**)	–	–	–	–	–	–	–	–	–	–	–	3.048
PhMeN (**16-II**)	–	2.816	–	–	–	–	–	–	–	–	–	–

Notes: (*a*) weak hy­dro­gen bond; (*b*) halogen bond.

**Table 4:** Axial bond lengths (Å) and bowl depths (Å) of the investigated BsubPcs Plane *a* is defined by the three pyrrole N atoms, plane *b* by the three imine N atoms and plane *c* by the six outermost C atoms.

Axial group	Axial bond length	Bowl depth to plane *a*	Bowl depth to plane *b*	Bowl depth to plane *c*
Cl (**1**)	1.884	0.580	1.116	2.456
Br (**2**)	2.052	0.568	1.101	2.457
MeO (**3**)	1.424	0.632	1.191	2.618
EtO (**4-I**)	1.421	0.641	1.193	2.598
EtO (**4-II**)	1.418	0.642	1.192	2.590
F_3_EtO (**5**)	1.437	0.631	1.188	2.468
ButO (**6**)	1.417	0.643	1.210	2.718
tButO (**7**)	1.415	0.660	1.225	2.711
OctO (**8-A**)	1.416	0.643	1.230	2.867
OctO (**8-B**)	1.421	0.646	1.209	2.652
OctO (**8-C**)	1.406	0.640	1.195	2.493
PhO (**9**)	1.441	0.624	1.181	2.607
Naphth­oxy (**10**)	1.441	0.620	1.202	2.881
Acetate (**11**)	1.473	0.603	1.158	2.389
Benzoate (**12**)	1.473	0.602	1.192	2.835
TMSO (**13**)	1.415	0.649	1.207	2.630
F_5_PhS (**14-A**)	1.955	0.587	1.183	2.751
F_5_PhS (**14-B**)	1.958	0.587	1.119	2.489
MePhS (**15-A**)	1.922	0.615	1.180	2.580
MePhS (**15-A**)	1.907	0.625	1.173	2.541
MePhS (**15-A**)	1.915	0.619	1.165	2.537
PhMeN (**16–1**, mol 1)	1.509	0.650	1.205	2.541
PhMeN (**16–1**, mol 2)	1.513	0.667	1.250	2.631
PhMeN (**16–2**)	1.513	0.659	1.217	2.595
